# Understanding the Role of Diethanolamine-Based Protic Ionic Liquids in Corrosion Inhibition: Electrochemical and Surface Characterization of Carbon Steel in Saline Environments

**DOI:** 10.1021/acs.langmuir.5c05642

**Published:** 2026-01-21

**Authors:** Caio Victor Pereira Pascoal, Mauro Andres Cerra Florez, Francisco Carlos Carneiro Soares Salomão, Eduardo Bedê Barros, Regiane Silva Pinheiro, Mohammad Rezayat, Gemma Fargas, Hosiberto Batista de Sant’Ana, Walney Silva Araújo

**Affiliations:** † Department of Metallurgical and Materials Engineering, 28121 Federal University of Ceará (UFC), Fortaleza, CE 60.440-900, Brazil; ‡ Ceara State University (UECE), Science and Technology Center, Fortaleza, CE 60.714-903, Brazil; § Department of Physics, Federal University of Ceará (UFC), Fortaleza, CE 60.455-760 Brazil; ∥ Department of Food Engineering, Federal University of Maranhão (UFMA), Imperatriz, Ma 65.915-060, Brazil; ⊥ Department of Chemical Engineering, Federal University of Ceará Fortaleza (UFC), Fortaleza, CE 60.440-554, Brazil; # Department of Materials Science and Engineering, (EEBE), 16767 Universitat Politècnica de Catalunya, (CIEFMA), UPC, Barcelona 08019, Spain; ∇ Barcelona Research Center in Multiscale Science and Engineering, Universitat Politècnica de Catalunya, UPC, Barcelona 08019, Spain

## Abstract

This study presents an evaluation of the corrosion inhibition behavior of three protic ionic liquids (PILs), 2-hydroxy diethanolamine formate (PIL A: 2-HDEAF), 2-hydroxy diethanolamine propionate (PIL B: 2-HDEAP), and 2-hydroxy diethanolamine pentanoate (PIL C: 2-HDEAPe), on A36 carbon steel in a chloride electrolyte (3.5 wt % NaCl). The emphasis was converged on elucidating interfacial adsorption, film formation, and surface chemistry that reinforce inhibitor efficacy. A complementary set of electrochemical and surface techniques, including weight loss measurements, potentiodynamic polarization, electrochemical impedance spectroscopy (EIS), optical microscopy, field-emission scanning electron microscopy (FE-SEM), and atomic force microscopy (AFM), was employed to evaluate the electrochemical response and characterize the inhibitor-modified steel surfaces. X-ray diffraction (XRD) was used to perform an identification of the main phases of corrosion products and adsorbed films. The adsorption behavior was quantitatively evaluated using several adsorption isotherm models, including Langmuir, Temkin, and Freundlich. Among them, the Frumkin isotherm provided the best description of the experimental data, yielding an average standard free energy of adsorption (Δ*G*°_ad_) of −20.89 kJ mol^–1^, which is indicative of predominantly physical adsorption at the steel/electrolyte interface. Among the PILs studied, PIL A exhibited the highest inhibition efficiency (>75%) and promoted the formation of a dense, protective interfacial film, whereas PILs B and C showed progressively lower performance. Inhibition efficiency correlated positively with inhibitor concentration and followed the trend PIL A > PIL B > PIL C. Surface morphologies demonstrated significant mitigation of chloride damage in the presence of PILs, consistent with electrochemical results. XRD analysis revealed the stabilization of surface films (iron oxides and oxyhydroxides), including goethite, which are indicative of altered interfacial reactions in the inhibited systems. These results accentuate the importance of interfacial adsorption evaluation and film formation mechanisms in governing corrosion inhibition performance, highlighting the potential of tailored PILs for surface protection in chloride-containing media.

## Graphical abstract

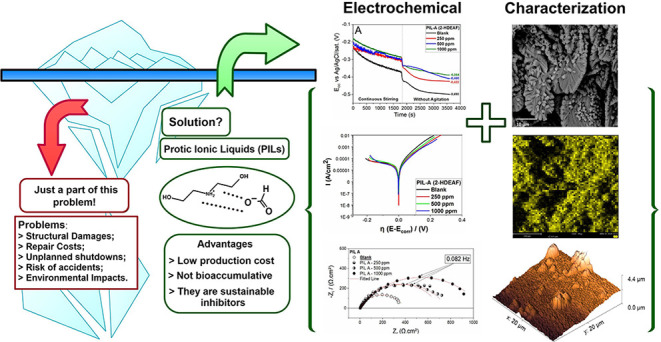

## Introduction

At first, corrosion continues to pose a substantial global challenge for society, with estimates from the Association for Materials Protection and Performance (AMPP) indicating annual losses exceeding US$2.5 trillion around 3.4% of the global GDP.^1,2^ Singularly, in the United States, corrosion-related costs surpass US$450 billion annually.

Beyond the economic burden, this issue compromises public safety and environmental integrity by accelerating infrastructure degradation.^3,4^ To combat these issues, industries increasingly adopt low-alloy steels due to their affordability and mechanical robustness. Implementing available corrosion control technologies could reduce costs by 15 to 35%, potentially saving US$375–875 billion worldwide each year. These preventive measures emphasize the necessity for proactive management across critical sectors.^1^ Consequently, in the face of this problem, the industry elects to utilize low-alloy as an alternative solution to mitigate corrosion-induced deterioration, mainly because of the low cost of production and higher structural resistance^5–8^

Industries have generally selected corrosion inhibitors as a combative method among the possibilities to face corrosion.^9^ The justificative is direct and simple. This method proves to be practical and efficient under varying conditions, particularly given that industrial operating parameters such as pH, temperature, and pressure can shift rapidly depending on production demands. Nevertheless, it is important to note that some corrosion inhibitors may pose risks to human health and the environment.^10,11^ Therefore, due to the need for new specific types of inhibitors in the oil industry that are technically safe, economically viable and environmentally friendly, ionic liquids (Aprotic and Protic) emerge as a plausible, innovative and sustainable option for the future of corrosion control.^12–15^ Aprotic Ionic Liquids (AILs) are particularly versatile due to their strong ionic character, resulting from the interaction between their cations and anions with various electrolytes, such as saline and acidic solutions.

In this context, a major advantage of these compounds is their reduced environmental impact, as they do not require toxic solvents, exhibit low volatility during synthesis, and possess high thermal stability. Nevertheless, some aprotic ionic liquids may be less environmentally favorable than protic ionic liquids due to their high chemical stability; for example, they can bioaccumulate in certain organisms and soils, thereby raising ecological concerns.^16,17^ Considering ecofriendly concerns associated with AILs, exploring alternative corrosion inhibitors is essential. Protic ionic liquids have emerged as promising alternatives, offering properties comparable to those of AILs, along with additional advantages such as simpler synthesis, lower cost, biodegradability, and minimal bioaccumulation.^18^ Considering these advantages, protic ionic liquids were the objects of study in this article, seeking to evaluate them as a promising solution in combating corrosion, balancing protective effectiveness with environmental responsibility, aiming at mutual awareness.^19^

According to recent studies on sustainable compounds,^20–25^ the growing relevance of protic ionic liquids (PILs) as corrosion inhibitors has been highlighted. This emphasis has been gaining momentum particularly due to their favorable environmental and sustainability profiles that have begun to be studied more in the past decade. Dissimilar conventional organic inhibitors, which are often volatile, toxic and poorly biodegradable, PILs can be molecularly engineered to exhibit low volatility, reduced ecotoxicity and key biodegradability attributes aligned with the principles of green chemistry justifying their choice as green inhibitors. Thus, their structural tunability allows the incorporation of environmentally benign cations and anions, including building blocks of natural origin or low toxicity, thus reducing the ecological impact during the synthesis and application phases^26–28^

In addition, PILs exhibit excellent thermal and chemical stability, ensuring long-term effectiveness in severe corrosive environments, such as chloride-rich media. This stability minimizes the frequency of reapplication and the associated waste generation. Importantly, the ability to tailor hydrogen bonding and proton donation characteristics improves their adsorption behavior and film formation efficiency on metal surfaces, which is critical for effective corrosion inhibition. From a sustainability perspective, many PILs are recyclable and reusable, contributing to resource efficiency and reducing overall environmental impact. Furthermore, their synthesis often requires milder conditions and fewer hazardous solvents compared to conventional inhibitors, further reinforcing their eco-friendliness. In view of the growing industrial demand for effective and environmentally responsible corrosion protection solutions, PILs emerge as a promising and innovative class of inhibitors. Their dual functionality seeks to combine technical performance with sustainability, thus positioning them as valuable alternatives in the development of more sustainable corrosion mitigation strategies.

In this context, the present article advances the investigation of PILs as corrosion inhibitors by introducing structurally modified compounds, distinguishing itself from most literature focused primarily on aprotic ionic liquids. The use of diethanolamine in the synthesis process enables the development of novel PIL-based inhibitors, a class still scarcely explored for corrosion protection applications.^29,30^ While maintaining the same metallic substrate (carbon steel) and corrosive medium (3.5 wt % NaCl) as in previous studies from the authors,^31,32^ this work differs significantly by incorporating complementary characterization techniques to provide a more comprehensive understanding of the chemical structure, electrochemical performance, and morphological features associated with corrosion.

This article proposals a detailed analysis of the corrosion products formed on the metal surface, contributing valuable insights into the inhibition mechanisms based on structural and surface-level evidence. Such discussions, commonly found in atmospheric corrosion literature, are rarely explored in the context of corrosion inhibitors, representing a novel contribution of this work. Although the corrosion inhibition performance of ionic liquids has been extensively reported in the literature, comparative studies focusing specifically on structurally related *protic* ionic liquids under identical experimental conditions remain limited. In this context, the present work addresses this gap by systematically evaluating a series of protic ionic liquids differing only in their acid moieties, enabling a direct correlation between molecular structure, adsorption thermodynamics, and electrochemical behavior.

By integrating open-circuit potential measurements, polarization studies, electrochemical impedance spectroscopy, adsorption isotherm analysis, and complementary surface and phase characterization, this study provides a comprehensive assessment of the inhibition mechanism and the evolution of interfacial films. Moreover, the emphasis on physisorption-dominated interactions and sustainability considerations offers new insights into the rational design and practical applicability of environmentally benign protic ionic liquids as corrosion inhibitors.

In sum, these methodological advances not only reinforce the originality of this study but also position it as a logical and necessary progression from earlier investigations, which focused primarily on mass loss, electrochemical impedance, and optical microscopy.^33,34^ The incorporation of structurally distinct bases in PIL synthesis significantly enhances the development of alternative and efficient inhibitors, underscoring the innovative and sustainable nature of this research within the framework of green chemistry and materials protection.

## Experimental Section

### Materials

To ensure the quality and reliability of the final products, the reagents were purchased from Aldrich (≥99% purity by mass), and the carboxylic acids were obtained from Sigma (99.6% purity by mass).

### Protic Ionic Liquid Synthesis

The protic ionic liquid synthesis was conducted with a dropping funnel by adding the reagents (bases and acids) in a three-necked glass flask equipped with a thermometer to determine the reaction temperature and a reflux condenser to avoid solvent evaporation. In summary, 2-hydroxy diethanolamine formate (PIL A = 2-HDEAF), 2-hydroxy diethanolamine propionate (PIL B = 2-HDEAP), 2-hydroxy diethanolamine pentanoate (PIL C = 2-HDEAPe) were used as chemical nomenclature.^35,36^ The reagents were purchased from Aldrich (≥99% purity by mass), and the carboxylic acids from Sigma (mass purity of 0.996) to ensure the quality and reliability of the final products.

It is important to note that, among the selected corrosion inhibitors, ethanolamine (ETA) and diethanolamine (DEA) are polar, hydrophilic, amine-based compounds with high solubility in water, primarily due to their ability to form hydrogen bonds. In aqueous sodium chloride (NaCl) solutions, both compounds remain fully soluble across a wide concentration range, owing to their inherent miscibility with water and compatibility with saline environments.^37^ Although the presence of NaCl does not significantly hinder their dissolution, the ionic strength and specific ion interactions can affect their physicochemical behavior particularly their protonation states, activity coefficients, and interactions with metal surfaces under corrosive conditions.

At a NaCl concentration of 3.5 wt % commonly used to simulate marine or physiological environments ETA and DEA exhibit good chemical stability and solubility.^38,39^ However, DEA may display slightly reduced mobility compared to ETA due to its larger molecular size and greater viscosity. The effective solubilization of these compounds is essential for their performance as corrosion inhibitors, as it enables their diffusion toward the metal substrate, where they can adsorb and form protective surface films. Additionally, the presence of both amine and hydroxyl functional groups facilitates interactions with chloride ions and metal cations, contributing to their adsorption behavior and inhibition efficiency.^40,41^

### Protic Ionic Liquids Characterization and Test Methods

Each mixture was prepared with a known mass of the protic ionic liquid and polar/apolar solvents (methanol/toluene ), and both were injected into a glass vial using a syringe. The contents were sealed with an aluminum cap, and a synthetic rubber plug in the vials. In addition, the space in the vials was minimized to avoid evaporation loss. The protic ionic liquids were injected into a DSA 5000 densimeter (Anton Paar, GRZ/Austria).

The measurements of the density and speed of sound at each temperature and atmospheric pressure of 100 kPa were obtained.^42^ DSA 5000 (Anton Paar) was used to evaluate pure liquids’ density and sound velocity values. The density (ρ) and viscosity (η) were measured at atmospheric pressure (101.325 kPa) in the temperature range of *T* = (293.15–333.15) K, using an Anton Paar SVM 3000 digital oscillation U-Tube, with the following standard uncertainties: *u*: *u*(*x*_1_) = 0.003, *u*(*T*) = 0.01 K, *u*(η) = 0.02 × η mPa·s, and *u*(ρ) = 0.0015 g·cm^–3^. The speed of sound was measured with the following standard uncertainties, *u*(*T*) = 0.01 K, *u*(*v*) = 0.9 m·s^–1^.

^1^H and ^13^C NMR (Nuclear Magnetic Resonance) experiments were performed utilizing an Avance DRX 500 spectrometer (Bruker, (RH/Germany), operating at 500 and 125 MHz for ^1^H and ^13^C equipped with an inverse detection One Probe. In addition, to assess the formation of the protic ionic liquid, PILs (30 mg) were dissolved in 0.5 mL of deuterium oxide (D_2_O) (99.9% D/Sigma), and the residual solvent peak was used as an internal reference (4.81 ppm) and analyzed in 5 mm tubes (Wilmad). For this examination, an infrared spectrometer (Fourier Transform Cary 630, Agilent, SC/USA) was used to evaluate liquid and solid samples.

Thus, the samples were applied directly to the spectrometer without prior preparation to avoid an error. Absorbance spectra were collected at a wavelength of the most significant interest for organic components (400–4000 cm^–1^) and with a spectral resolution of 1 cm^–1^. TGA (Thermogravimetric Analysis) analysis was performed for all protic ionic liquids to investigate the thermal stability of the materials under N_2_ environment conditions using SDTA 851 (Mettler Toledo, ZRH/Switzerland). All the PILs exhibited similar behavior under a nitrogen atmosphere in the temperature range of 30 to 400 °C, with a flow rate of 50 mL/min and a heating rate of 10 °C/min. Finally, detailed information (tables, figures, and discussion) is provided in Supporting Information.

### Sample Preparation for General Evaluations

The chemical composition of the structural steel used in the experiments (wt %) was determined with structural steel A36 ASTM (CMC Committals steel) (USA) utilizing PDA 7000 Optical Emission Spectrometer model Shimadzu (Kyoto, JP), with average: C = 0.21029%, Si = 0.03306%, Mn = 0.50905%, P = 0.00569%, S = 0.00841%, Ni = 0.02425%, Cr = 0.0233% and Fe = 98.71%, similar composition to the literature data.^43^ First, the steel samples (A36) with a size of 0.5 cm^2^ were ground with 120, 220, 400, 600, and 1200 grit every paper without further polishing.

Before electrochemical evaluation, the material was washed with distilled water and ethanol. Then, the samples were dried with the assistance of a heat gun to ensure that all the solvents used in cleaning were removed.

### Corrosion Tests

#### Weight Loss Measurements and Corrosion Evaluation by Immersion Measurements

ASTM A36 carbon steel specimens (1.0 cm × 3.5 cm × 0.5 cm) were primed by the procedure (abrade, rinse, dry) to first weight (W1). The immersion test was conducted in 500 mL of 3.5 wt % sodium chloride (NaCl) solution, with and without 1000 ppm of PILs (A–C), for a duration of 100 h. The solution volume was calculated based on the total surface area of the samples, following the ASTM G1 standard for the preparation, cleaning, and evaluation of corrosion test specimens.^44^ In detail, the immersion tests were conducted in a 100 mL solution containing 3.5% by weight sodium chloride (NaCl), the concentration of which was determined based on the total area of the sample. Tests were performed with and without PILs with two specific concentrations, 500 and 1000 ppm, for 24 h. Furthermore, the objective is to evaluate samples that were exposed to the protic ionic liquids (PILs) 01 (2-HEAF) and 02 (2-HDEAF), which were previously identified as the most effective corrosion inhibitors based on electrochemical tests and mass loss measurements.

#### Electrochemical and Conductivity Measurements

Electrochemical measurements were conducted operating a conventional three-electrode cell configuration, in accordance with the format adopted in the authors’ previous publications.^31,32,45,46^ The working electrode consisted of A36 carbon steel with dimensions of 0.5 cm × 0.5 cm was used as the specimen size standard. All electrochemical measurements were conducted under naturally aerated conditions, without intentional deaeration or oxygen control. This experimental choice was made to better represent practical saline environments and to ensure consistency across all measurements. Under these conditions, the dissolved oxygen content corresponds to ambient laboratory conditions for aqueous 3.5 wt % NaCl solutions at room temperature. A platinum wire (1.2 cm × 1.2 cm) functioned as the counter electrode, while a KCl-saturated Ag/AgCl wire was used as the reference electrode, chosen for its stability under varying temperatures and potential water evaporation. This evaluation was possible with Autolab 302N Modular potentiostats/galvanostats (UT/Netherlands). The samples were immersed in solutions containing protic ionic liquids (250, 500, and 1000 ppm) for electrochemical evaluation in saline solutions.

Electrochemical impedance spectroscopy (EIS) measurements were carried out over a frequency range from 100 kHz to 0.01 Hz using a sinusoidal perturbation with an amplitude of 20 mV after 3600 s of stabilization at the open circuit potential (OCP). Polarization curves were subsequently recorded within a potential window of ± 200 mV relative to the OCP at a scan rate of 1 mV s^–1^, with a current limit of 1 mA, following the same OCP stabilization period.

The scan rate of 1 mV s^–1^ was selected to ensure quasi-steady-state conditions during polarization, in this manner minimizing capacitive contributions and enabling accurate determination of corrosion parameters.^47,48^ This value is widely adopted in the literature for corrosion studies involving protic ionic liquids as inhibitors, particularly to maintain consistency and enable meaningful comparison with previously reported results.^49^

The potential range selected for the polarization measurements (from −0.2 to 2.0 V vs OCP) was carefully chosen based on well-established electrochemical protocols for evaluating corrosion inhibitors in saline environments. This range is commonly employed in the literature to ensure coverage of both the cathodic and anodic domains relevant to carbon steel corrosion, thereby allowing for a thorough assessment of inhibitory performance^50,51^ (Table 1).

**Table 1:** Comparison of Electrochemical Parameters Used in Corrosion Inhibition Studies with Ionic Liquids

Study (Metal/Electrolyte)	Polarization Range vs OCP	Scan Rate/Perturbation	EIS Frequency Range	Key References
This work (A36 steel/3.5 wt % NaCl)	–0.2 V to +2.0 V	1 mV/s scan; 20 mV AC	100 kHz–0.01 Hz	DEA (Diethanolamine)-PILs (Protic Ionic Liquids)
AA 6061 Al/1 M HCl with BMIm IL	Approx −0.25 to +1.5 V	∼1 mV/s	100 kHz–0.01 Hz	Imidazolium IL study^53^
Mild steel/2 M HCl with EMIm, BMIm, HMIm ILs	±0.8 V around OCP	∼1 mV/s	100 kHz–0.01 Hz	Imidazolium ILs^54^
Mild steel/1 M HCl with imidazolium ILs	Polarization ±0.8 V around OCP	1 mV/s typical	100 kHz–0.01 Hz	Smart IL inhibitors^55^
Mild steel/1 M HCl with R_8_-, R_10_-, R_12_- imidazolium ILs	PDP ± 1 V around OCP	Standard PDP	100 kHz–0.01 Hz	R_n-IL series^56^
Carbon steel/0.5 M HCl with C_2_-/C_10_-task-specific ILs	±1 V around OCP	Not specified (∼1 mV/s)	100 kHz–0.01 Hz	Corrosion Science 2018, Task-specific ILs^57^

It enables the detection of critical electrochemical phenomena such as hydrogen evolution at cathodic potentials and active metal dissolution and passivation behavior at anodic potentials.^52^ These regions are essential to elucidate the mechanism of inhibitor action, especially for compounds like protic ionic liquids (PILs), whose adsorption and protective behavior may vary across different polarization regimes.

The use of open circuit potential (OCP) as a reference ensures that the system has reached a quasi-steady-state electrochemical condition after 3600 s of immersion, minimizing transient effects and enhancing data reproducibility. Moreover, the amplitude of 20 mV and frequency range from 100 kHz to 0.01 Hz used in the electrochemical impedance spectroscopy (EIS) experiments align with standard practices in corrosion science for resolving processes occurring at the metal–solution interface and characterizing protective film formation.

Therefore, the potential window and experimental parameters were selected not arbitrarily but in accordance with widely accepted methodologies in the field, ensuring the scientific validity and relevance of the results to the study of corrosion inhibition by PILs.^13^

The electrochemical parameters adopted in the present study (Topic 1) are in strong agreement with those reported in the literature (Topics 2–5), supporting the scientific validity of the methodology. The polarization potential ranges from −0.2 to 2.0 V versus OCP ensures coverage of both cathodic and anodic processes, a practice consistently observed across various studies employing ionic liquids (ILs) as corrosion inhibitors. For instance, similar intervals were used by some authors in literature (Table 1), who explored task-specific ILs in chloride and acidic media. This consistency highlights the reliability of these ranges for assessing inhibitor behavior on carbon steel substrates.^58,59^

Moreover, the use of a 20-mV sinusoidal perturbation and a frequency range from 100 kHz to 0.01 Hz in EIS analysis, as employed in this article, aligns well with established corrosion science protocols. Comparable ranges and amplitudes were reported by authors, emphasizing their adequacy for evaluating surface film formation and charge transfer mechanisms. Additionally, referencing OCP after 3600 s of immersion ensures electrochemical stabilization, a procedure likewise implemented in the comparative studies. Collectively, these methodological consistencies reinforce the suitability of the chosen parameters, confirming they are not only aligned with the state of the art but also effective for generating reliable, reproducible data in corrosion inhibition studies using IL-based systems. The critical micelle concentration (CMC) of protic ionic liquids was determined by plotting the conductivity data against the (PILs) concentration at 25 °C. The solution was added dropwise with a micropipette (5 μL), assisted by magnetic stirring (200 rpm) to dissolve the electrolyte with the assistance of the pH/conductometer (Metrohm/model 914), (UT/Netherlands).

The CMC value was determined when the solute addition did not change the conductivity value during a specific period (stability phase). This material’s numerical data were based on the analyses’ arithmetic mean and mean deviation.^60^

#### Surface Analysis

Surface evaluation was possible with the aid of optical microscopy. The sample’s surface was assessed in a saline solution containing 1000 ppm of all PILs for 24, 48, and 72 h. Scanning Electron Microscopy (SEM) was performed using equipment Quanta 450-FEG (FEI) (Thermo Fisher Scientific), (OR/USA) to promote a detailed material evaluation.

The methodology applied in the ASTM G1 standard practice for preparing, cleaning, and evaluating corrosion test samples was followed. Atomic Force Microscopy (AFM) was utilized to study the changes in the surface morphology of A36 carbon steel after 24 h of immersion at 298 K for surface analysis.

The AFM measurements were obtained in the intermittent contact mode with Asylum MFP-3D BIO equipment (SB/USA) along with curved radius tips smaller than 10 nm and a resonant frequency of 75 kHz. The scan area of AFM imaging is at least 10 μm × 10 μm. Finally, the corrosion products were evaluated by applying the X-ray diffraction method, where steel (A36) was immersed for 30 days in an electrolyte solution with a fixed concentration of inhibitor (1000 ppm) and without inhibitor as a comparison of the steel surface after the period (30 days).

Then, the former corrosion product was filtered and evaluated using an X-ray diffractometer (RX/DMAXB, Rigaku, TKY/Japan). The carbon steel specimens exposed to the blank (3.5 wt % NaCl) and inhibitors (PILs) for 24 h at 298 K were selected for the morphological studies.

For a more complete assessment of the surface after immersion, a scanning electron microscope (SEM) Thermo-Phenom, Model: G5 XL, MA, USA) was operated on for morphological examination. In detail, this equipment was attached to Energy Dispersive Spectroscopy Systems (EDS) from Oxford Instruments (Abingdon, UK) for the compositional analysis of surface corrosion products with a focus on the elemental proportion found on the surface.

## Results and Discussion

### Anticorrosive Performance of PILs

#### Weight Loss Measurements

Mass loss is a fundamental technique for evaluating corrosion inhibitors, as it provides a direct and quantitative assessment of metal degradation over time under simulated real-world conditions. The corrosion rate is determined by measuring the mass of a sample before and after exposure to a corrosive environment, typically a saline solution, enabling a precise comparison of inhibitor performance. This method is particularly valuable for long-term studies as it captures the cumulative effects of corrosion and serves to validate electrochemical techniques such as potentiodynamic polarization and electrochemical impedance spectroscopy. Its simplicity, cost-effectiveness, and broad applicability make it a widely adopted tool in research and industrial settings, ensuring reliable and comparable data for corrosion protection strategies.^61–63^

In sum, mass loss assays were performed by adding each of the investigated protic ionic liquid (PIL) inhibitors at concentrations of 250, 500, and 1000 ppm. The ASTM A36 carbon steel coupons (1.0 × 3.5 × 0.5 cm) underwent priming through the following procedure: abrading, rinsing, and drying, resulting in the initial weight (W_1_). The results show a significant decrease in corrosion rate (υ_corr_) with the addition of PILs, indicating special protection of carbon steel under a saline environment, as shown in Table 2. PIL A exhibited a typical behavior in the study of corrosion inhibitors, where its concentration was raised to the maximum value of 1000 ppm, and the inhibition efficiency continued to increase. Subsequently, after the immersion exposure, the specimens were cleaned to remove corrosion products utilizing Clark solution (admiring the ASTM G1 standard methodology), and the samples were then dried and reweighed (W_2_). Weight loss was determined by gravimetric tests using an analytical balance, Shimadzu (220 g) with 0.0001 g precision.

**Table 2:** Weight Loss Corrosion Parameters of Carbon Steel (100 h)^a^

250				500			1000		
PIL	A/Δ*W*	υ_corr_ (mmy)	%IE	A/Δ*W*	υ_corr_ (mmy)	%IE	A/Δ*W*	υ_corr_ (mmy)	%IE
Blank	0.07 ± 0.01	1.31	0	0.07 ± 0.01	1.3141	0	0.07 ± 0.01	1.3141	-
PIL A	0.02 ± 0.01	0.64	50.3	0.02 ± 0.006	0.53	58.65	0.01 ± 0.03	0.32	75.05
PIL B	0.03 ± 0.02	0.75	52.0	0.02 ± 0.01	0.45	71.42	0.03 ± 0.01	0.88	44.57
PIL C	0.03 ± 0.01	0.68	57.1	0.05 ± 0.003	0.50	68.27	0.01 ± 0.02	0.86	45.50

a A—average; W—weights 1 and 2; υ corr—corrosion rate; SD—standard Deviation.

The weight loss corrosion parameters summarized in Table 2 clearly demonstrate the substantial influence of protic ionic liquids (PILs) on the corrosion behavior of carbon steel following 100 h of exposure in a saline environment. Across all tested concentrations (250, 500, and 1000 ppm), the incorporation of PILs led to a marked reduction in both mass loss (Δ*W*) and corrosion rate (υ_corr_) when compared to the uninhibited (blank) sample. At 250 ppm, PIL-C achieved the highest inhibition efficiency (57.1%), followed closely by PIL-B (52.0%) and PIL-A (50.3%).

This inhibition trend improved further at 500 ppm, where PIL-B and PIL-C exhibited superior efficiencies of 71.4% and 68.2%, respectively, while PIL-A reached 58.6%. These results suggest that the protective effect of the PILs becomes more pronounced with increasing concentration, up to an intermediate range, particularly for PIL-B and PIL-C, indicating enhanced surface coverage and adsorption at moderate concentrations.

At the highest concentration tested (1000 ppm), however, only PIL-A maintained a significant improvement in performance, attaining an efficiency of 75.05%.

In contrast, PIL-B and PIL-C displayed a considerable decline in their protective capabilities, with inhibition efficiencies decreasing to 44.57% and 45.50%, respectively. This reduction at elevated concentrations may be attributed to factors such as the aggregation of inhibitor molecules in the solution or the saturation of active sites on the steel surface, both of which can impede the formation of a coherent and protective adsorbed film.

Overall, the weight loss results highlight the concentration-dependent behavior of these PILs and underscore the critical role of molecular structure in determining inhibition performance. These findings emphasize the importance of optimizing both concentration and molecular design parameters to maximize the corrosion protection efficiency of PIL-based inhibitors in chloride-rich environments.

Protic ionic liquids B and C showed an unfavorable result. The inhibitory effect decreased when the concentration was increased to the maximum value. The increased carbon chain in the compounds’ structure^64^ probably explained this result.^64^ In the weight loss tests, a maximum efficiency of 75.05% was obtained at a concentration of 1000 ppm in the saline electrolyte. This result proves the importance of evaluating the effects of structural modification of corrosion inhibitor molecules. In the case of protic ionic liquids, modifying the carbon chain did not yield the favorable results commonly reported in the literature.^12,65^ This process was systematically repeated for weight loss measurements.^44,66^

Additionally, the same methodology was replicated for mass losses with heat, utilizing two optimized concentrations (250, 500, and 1000 ppm). Each experiment was conducted in triplicate. The weight loss (ΔW) was determined using the following equation, consistent with the literature on mass-loss measurements.^9,67–70^

1 $$\Delta W=W_{1}-W_{2}$$

The corrosion rate, denominated by υ_corr_ (mmy), was calculated using eq 2:^13,15,66,71–74^

2 $$\upsilon corr=\frac{\Delta W*k}{S.t.p}$$

Fundamentally, the symbol (*S*) is the sample area (cm^2^), (*t*) is the dousing period (*h*), and (*p*) is the density (*k*). The inhibition efficiency, % IE_WL_ was calculated by

3 $$\%IE=\frac{\upsilon \mathrm{corr}\left(b\right)-\upsilon \mathrm{corr}\left(i\right)}{\upsilon \mathrm{corr}\left(b\right)}x100$$

Where υ_corr(b)_ and υ_corr(i)_ are the corrosion rates with inhibitors and without them.^13,15,66,71–74^ After this discussion, the weight loss measurements demonstrated that the evaluated protic ionic liquids (PILs), designated as B and C, exhibited relatively low inhibition efficiencies under the tested conditions. Although a moderate maximum efficiency of 75.05% was recorded at the highest concentration (1000 ppm) in the saline environment, the overall inhibitory performance declined with increasing concentration, suggesting nonlinear and concentration-dependent behavior.

Such a trend is often attributed to structural limitations inherent to the molecular architecture of the PILs, particularly the influence of the carbon chain length in their chemical structure, as highlighted in previous studies. The experimental methodology strictly followed established standards for weight loss assessments, incorporating triplicate measurements and a range of concentrations (250, 500, and 1000 ppm), thereby ensuring the reproducibility and reliability of the obtained data.

The inhibition efficiencies, calculated based on mass loss reduction, clearly reflected the limited protective capacity of the tested compounds when exposed to aggressive chloride-rich media. In comparison with the literature, the inhibition performance of these PILs was notably inferior to that of other ionic liquids with more optimized molecular designs. PILs containing shorter alkyl chains or aromatic functional groups typically achieve superior efficiencies, frequently surpassing 90% under similar saline conditions.^40,75,76^

These findings underscore the critical role of molecular engineering in the development of effective corrosion inhibitors, particularly for saline environments. It becomes evident that simple carbon chain modifications, as applied in the present study, are insufficient to achieve enhanced inhibition performance. This outcome corroborates recent investigations on task-specific ionic liquids (TSILs), which emphasize that strategic modifications at both the cationic and anionic moieties are fundamental to improving adsorption characteristics and overall corrosion protection.^41^

#### Adsorption Isotherm and Thermodynamic Analysis

The determination of adsorption isotherms for corrosion inhibitors relies fundamentally on accurate and representative data reflecting the true extent of metal-inhibitor interactions under corrosive conditions. Among the various techniques available, weight loss measurements are widely regarded as the most reliable and practical method for this purpose.

This is because weight loss experiments simulate real-world exposure conditions, where metal samples are immersed for extended periods in corrosive media, allowing continuous interaction between the inhibitor and the metal surface. The resulting data directly quantifies the total material loss due to corrosion over time, making it an integral and cumulative measure of inhibitor performance. This time-integrated response reflects both kinetic and thermodynamic aspects of the adsorption process, providing a robust data set suitable for fitting and interpreting adsorption isotherms. Moreover, the weight loss method offers distinct advantages over electrochemical techniques when evaluating adsorption behavior.

Unlike electrochemical methods that often involve short-term measurements and may reflect only surface processes at specific time points, weight loss testing encompasses the full corrosion process over prolonged exposure, capturing variations in inhibitor adsorption and desorption dynamics under realistic operational conditions. This comprehensive approach yields corrosion rate data that is both quantitative and reflective of actual material degradation, ensuring that the adsorption parameters derived from isotherm models truly represent the inhibitor’s performance in field-like environments.

Therefore, when the objective is to construct accurate adsorption isotherms that reflect the inhibitor’s real protective capacity, weight loss measurements remain the most appropriate and scientifically robust technique (Figure 1). The protective effect of corrosion inhibitors is strongly influenced by their ability to be adsorbed on the surface of metallic materials such as carbon steel. Usually, corrosion inhibitors are adsorbed on the surface by physical or chemical adsorption processes; this reaction/interaction creates a protective layer that reduces the corrosive attack.^77,78^ Various isotherm models can be applied to identify the most suitable adsorption isotherm, enabling the characterization of the interaction between corrosion inhibitors and the metal surface. This interaction, governed by organo-electrochemical reactions, provides key insights into the inhibition mechanism. The Langmuir, Temkin, Freundlich, and Frumkin isotherms have been verified, and all these equations have been reported in the literature about the adsorption of chemical compounds. All these isotherms are of the general form (eq 2):^79^

4 f(θ,x)exp(−2aθ)=KC

**Figure 1:**
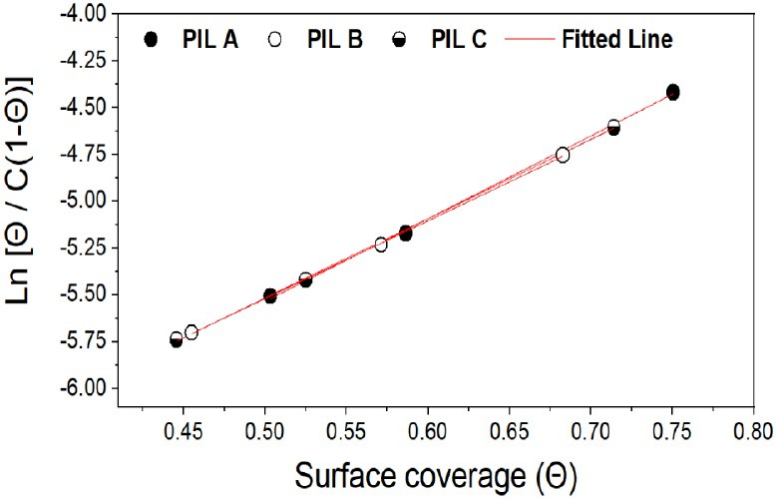
Frumkin adsorption isotherm model for PILs adsorption at the NaCl interface using the weight loss method.

The factor *f*(*θ,x*) represents the configuration variant, which varies according to the physical model and the assumptions used in the derivation of the isotherm.

The variable theta (θ) represents surface coverage, calculated as θ = I.E/100, where I.E. (inhibitory efficiency) reflects the average efficiency results obtained via the weight loss technique. Additionally, (*C*) stands for the inhibitor concentration in 100 mL of solution, while (*a*) denotes the lateral interaction term, describing molecular interactions within the adsorption layer. The variable (*K*) represents the adsorption–desorption equilibrium constant. In this study, the Frumkin adsorption isotherm (eq 3) demonstrated a good fit with the experimental data.^79^

5 $$KC=\left[\frac{\theta }{1-\theta }\right]\exp\left(-2a\theta \right)$$

The adsorption isotherms showed a good fit with the experimental data, as indicated by the high correlation coefficient (*R* ^2^) values. In particular, the Frumkin isotherm (Figure 1) demonstrated strong agreement with the observed results. The R^2^ values obtained were: PIL A = 0.99907, PIL B = 0.99974, and PIL C = 0.99962.

For the Langmuir model, these values were PIL A = 0.98909, PIL B = 0.95369, and PIL C = 0.96919. In the case of the Temkin isotherm, they were PIL A = 0.9655, PIL B = 0.08261, and PIL C = 0.26128. Finally, for Freundlich, the values recorded were PIL A = 0.98195, PIL B = 0.1167, and PIL C = 0.31359. Distinct variations in linear coefficients were observed across the evaluated models (Frumkin, Langmuir, Temkin, and Freundlich).^1^

The results indicated that the Frumkin isotherm model was the most suitable for analyzing the system data. This model enhances the Langmuir isotherm by reducing specific parameters, offering a more refined correlation between the adsorbed surface density and the concentration of the chemical species in solutions. During this process, a monolayer coating forms; however, according to studies on corrosion inhibitors, such coatings are often considered suboptimal in saline environments.

These inhibitors permit undesirable interactions, such as attractive forces between chains or repulsive forces between polar groups, that should ideally occur only between adjacent molecules. These interactions lead to “weak points” within the coating on the metal surface, making the substrate more prone to corrosion. Although this type of inhibitor offers a certain level of protection, its effectiveness is significantly lower compared to other inhibitors, which, according to the literature, can achieve efficiency levels exceeding 95%.

It is important to point out that the choice of the isotherm that best fits the results provides crucial answers for the study of corrosion on the behavior of the inhibitor, such as the coverage of the unit at different concentrations and the lateral interaction between the molecules of the same. The free energy of adsorption (Δ*G*°_ads_) was calculated from the Frumkin isotherm values, which demonstrated the best correlation with experimental data (eq 6), where (*T*) is the experimental temperature in Kelvin, (*R*) is the universal gas constant, and 55.5 represents the water concentration in moles.^80^ Also, the adsorption–desorption equilibrium constants (*K*_ads_) were determined to be 0.0004310 for PIL A, 0.0004825 for PIL B, and 0.0005013 for the PIL C unit of measurement (L.mg^–1^), and (MM) as the molar mass of the corrosion inhibitor.

6 $${\Delta G}^{0}ads=-\mathrm{R.T.}\ln\left(K_{ads}.MM.10^{3}.55,5\right)$$

The lowest values of *K* were related to low interactions between the inhibitor molecules and the metal surface by the electrolyte solution (NaCl 3.5wt %).^79^ This result confirms that the nature of inhibition occurs physically in the electrochemical layer, and interestingly, the strength of this interaction decreases with increasing temperature. When comparing the inhibition levels achieved by the studied corrosion inhibitors, an important consideration should be highlighted in this section. Although the numerical values of inhibition efficiency are relatively modest, they should not be overlooked or disregarded, as is sometimes observed in literature. Every experimental result, regardless of its magnitude, carries scientific relevance and merits careful analysis and discussion.

Even when the primary focus shifts from efficiency toward other aspects, such as environmental sustainability or the development of greener alternatives, the data provides valuable insights that contribute to advancing knowledge in the field of corrosion science. In sum, they are leading to Δ*G*°_ads_= −20.28 (PIL A), −20.97 (PIL B), and −21.42 (PIL C) by kJ mol^–1^, with an average of 20.89 kJ mol^–1^. The literature standard is that negative values of Δ*G*°_ads_ denote adsorption via a spontaneous process, which is important for understanding the corrosion inhibition strength and mechanisms.^13,14,81^ Evaluating adsorption data such as Gibbs Free Energy is essential because these results can determine the nature of the process between the metal surface and the inhibitor in the corrosion investigation. It is accepted that Δ*G*°_ads_ values near −20 kJ/mol have lower adsorption energies, suggesting that the electrostatic interaction between charged molecules and the material, known as adsorption by physisorption, can be explained by the ionic nature of the compound. In agreement with the literature on corrosion inhibitors, the mean value obtained of −20.89 kJ mol^–1^ was close to that found by several authors who investigated inhibitors by physical adsorption, such as Zeng et al.^82^ with values of Δ*G*°_ads_ −20.0 (kJ mol^–1^) and Subasree.^83^ with results from Δ*G*°_ads_ −16.0 to −19.0 (kJ mol^–1^).

#### Open Circuit Potential Measurements

According to the literature, monitoring the open circuit potential (OCP) as a function of time is a valuable technique for assessing interfacial changes at the metal–electrolyte boundary during corrosion processes.^14,84^ In the present study, the electrochemical system was subjected to two sequential hydrodynamic conditions: (i) forced convection (stirring phase) and (ii) stationary conditions (rest phase). The stirring was maintained for 1.800 s (30 min) to promote uniform mass transport and ensure homogeneous exposure of the metal surface to the electrolyte.

This agitation was designed to simulate dynamic conditions that mimic practical engineering systems, where electrolyte flow influences mass transfer and corrosion rates. After this initial period, agitation was stopped for an additional 1,800 s to allow the system to reach electrochemical stabilization under quiescent (stationary) conditions, which better represent the thermodynamic stability of the corrosion process.^85^ The choice of the 1,800-s interval for both agitation and stationary phases was based on preliminary tests and in line with protocols reported in previous studies evaluating stabilization time for open circuit potential measurements in similar saline environments.^85^ During the stirring phase, the Reynolds number (Re) was estimated at approximately 3,500, classifying the flow as turbulent, according to the standard definitions for electrochemical cells with rotational or mechanical stirring. The stirring facilitated constant electrolyte renewal at the metal interface, minimizing concentration gradients and enhancing surface activation. Upon cessation of agitation, the system transitioned to natural diffusion-controlled conditions, allowing the observation of the true thermodynamic potential of the steel-electrolyte interface without the influence of convective mass transport. Figure 2 shows the time-dependent OCP profiles obtained for the carbon steel samples immersed in 3.5 wt % NaCl solutions, both in the absence (blank) and presence of PILs at varying concentrations. A clear potential shift toward more positive values was observed for all PIL-containing systems when compared to the blank (−0.498 V vs Ag/AgCl). The final stationary potentials (after 1.800 s under static conditions) for the most representative concentrations were recorded as follows: −0.384 V (PIL-A at 1000 ppm), −0.375 V (PIL-B at 500 ppm), and −0.396 V (PIL-C at 250 ppm).

**Figure 2:**
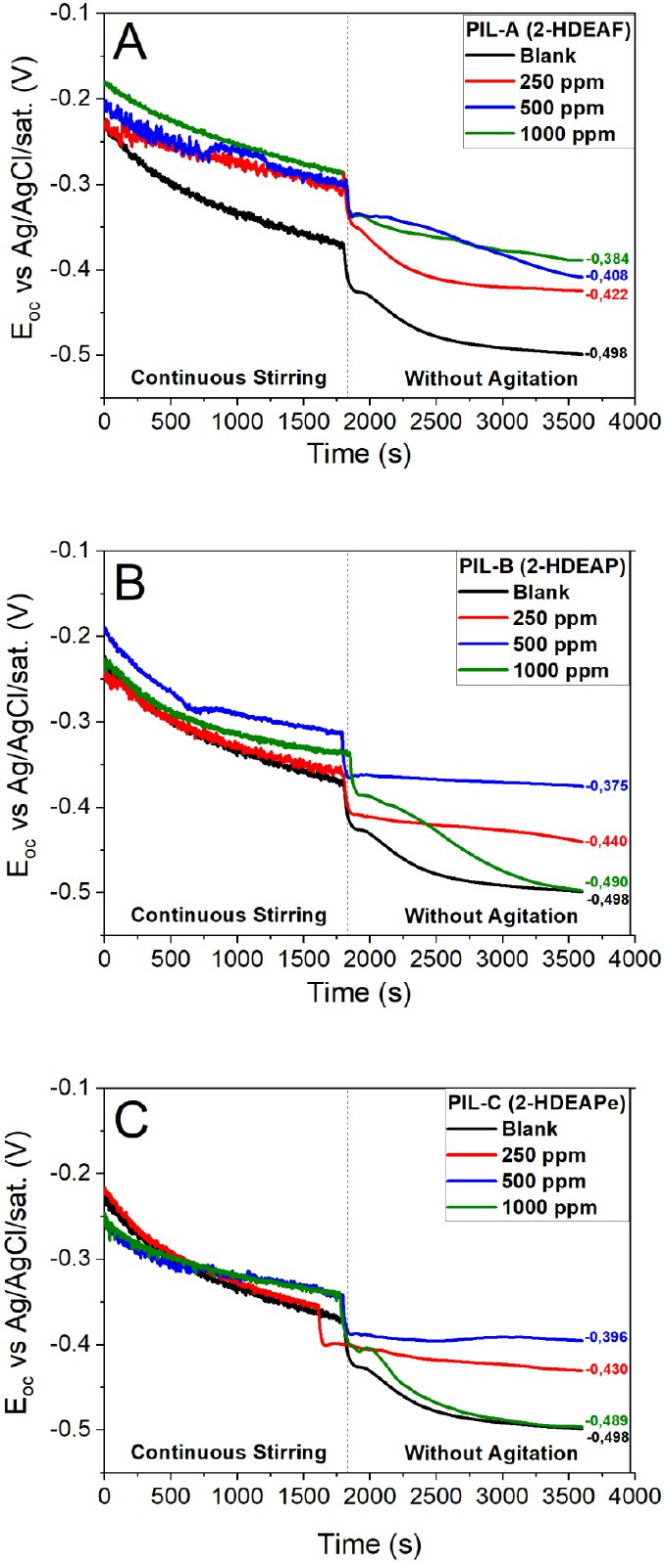
Open circuit potential (OCP) evolution of A36 carbon steel immersed in 3.5 wt % NaCl solution in the absence and presence of protic ionic liquids: (A) PIL A (2-HDEAF), (B) PIL B (2-HDEAP), and (C) PIL C (2-HDEAPe), highlighting the differences in OCP behavior over the monitored time interval.

This behavior is associated with the adsorption of inhibitor molecules onto the steel surface, forming a barrier layer that alters the metal/electrolyte interfacial properties. To further address the influence of hydrodynamic conditions, the Reynolds number during the weight loss tests was also considered. Based on solution properties and agitation parameters, the NRe was estimated at approximately 3,500 for both electrochemical and gravimetric experiments, ensuring consistency between test methodologies. Although this study did not perform a direct OCP versus Reynolds number analysis (as that was not the primary objective), the controlled application of turbulent flow followed by a stationary period was purposefully designed to simulate realistic service conditions and ensure reproducible and thermodynamically meaningful OCP values. This experimental design aligns with recent studies that emphasize the importance of hydrodynamic control for corrosion inhibitor evaluations.

#### Potentiodynamic Polarization Curves

Polarization curves were determined from different concentrations (250, 500, and 1000 ppm) of the three protic ionic liquids on the potentiodynamic behavior of A36 carbon steel in a 3.5wt % NaCl solution at room temperature (Figure 3).

**Figure 3:**
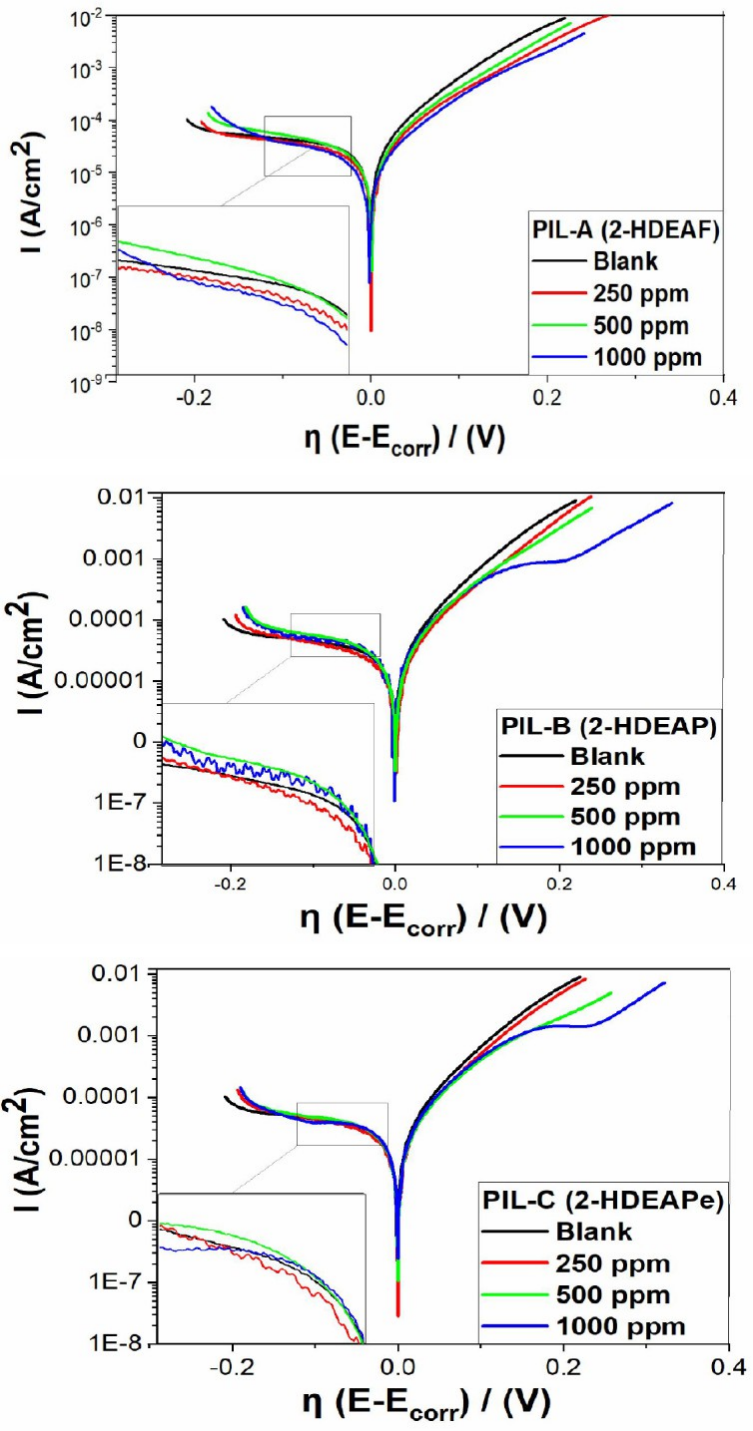
Overpotential plots of potentiodynamic polarization curve for different concentrations of PIL A.

The polarization results indicate that the addition of protic ionic liquids to the solution influences the corrosion reaction mechanism. This effect is observed on both the cathodic and anodic branches of the polarization curves, qualitatively demonstrating the inhibitory capacity of the ionic liquids. Typically, the polarization curves yield a corrosion potential value, which is used as a reference to plot the overpotential. This facilitates the analysis of polarization behavior, starting from the cathodic region (−0.2 V) and extending to the anodic region (2 V), at a constant sweep rate of 1 mV/s.

Figure 3 shows the results of all inhibitors (PIL A, B, and C), where it is possible to observe that the ramifications of the anodic fraction tend to have smaller values of current density (A/cm^2^) when compared to the test without inhibitor (blank) and thus are characteristic of an anodic type of inhibitor owing to this qualitatively observed difference. In addition, the current values for all the investigated compounds (PILs) were lower than the blank value at all concentrations (250 to 1000 ppm), confirming the anodic type of protection.

Furthermore, the *E*_corr_ values of the ASTM A36 work electrode/electrolyte in the presence of PILs shifted by approximately 90 mV in Ag/AgCl as a reference electrode saturated with KCl. This final average value indicates that, compared to the blank samples, the corrosion potential changes to more positive values (anodic direction) are mainly due to the polarization of the anodic reaction after the *E*_corr_ point. In summary, this shift was associated with the alteration of the substrate surface (ASTM A36 carbon steel) owing to the adsorption process of the inhibitor. In electrochemistry, the classification of a compound as an anodic or cathodic corrosion inhibitor is variable, depending on the technique chosen for evaluation.

The combination of potentiodynamic polarization and open circuit potential (OCP), for example, provides critical data for the assessment of inhibitors, where when the displacement observed in the OCP values was greater than 85 mV about the value obtained by the blank (without the addition of inhibition), it is proved that with the addition in the system, the potential tended to have positive or negative values, thereby preliminarily categorizing the type of inhibitor.

To contextualize, in studies of El-Tabei, El-Tabey, and Basiony.^86^ With diatonic surfactants, chemical compounds with structures analogous to those of ILs, as a corrosion inhibitor in 1 M HCl electrolyte, the value of the shift was in the range of ± 85 mV in both branches (anodic and cathodic), characterizing a mixed type of inhibitor.

The study by Verma et al.^24^, which investigated newly synthesized ionic liquids (2,4-diamino-5-(phenylthio)-5H-chromeno­[2,3-*b*]­pyridine-3-carbonitriles) as corrosion inhibitors in a 1 M HCl electrolyte, reported a maximum shift in the corrosion potential (*E*_corr_) of 75 mV below the 85 mV threshold commonly used to classify inhibitors as anodic or cathodic. The results obtained in the present study using protic ionic liquids (PILs) are consistent with values reported in the corrosion literature. Specifically, polarization curves demonstrated a reduction in current densities upon the addition of the PILs, indicating their inhibitory effect. These findings support the evaluation and application of these compounds as potential electrochemical corrosion inhibitors in saline environments, particularly in 3.5% NaCl solutions that simulate marine conditions. As shown in Table 3, the addition of PILs A, B, and C resulted in noticeable changes in the characteristic polarization behavior, including a significant decrease in current density, thereby enhancing the protection of the material in the corrosive medium.^87–89^

**Table 3:** Polarization Parameters of A36 Carbon Steel in 3.5% NaCl without and with Different Concentrations of PILs^a^

	*C* (ppm)	*E*_corr_	*I*_corr_	η
Blank	-	–0.554 ± 0.020	9.51 ± 0.34	-
PIL A	250	–0.428 ± 0.003	5.28 ± 0.49	44.42
	500	–0.453 ± 0.033	4.00 ± 0.21	57.94
	1000	–0.415 ± 0.013	2.37 ± 0.87	75.11
PIL B	250	–0.518 ± 0.010	5.66 ± 0.90	40.45
	500	–0.403 ± 0.025	2.91 ± 0.10	69.38
	1000	–0.503 ± 0.037	5.94 ± 0.51	37.54
PIL C	250	–0.433 ± 0.009	4.55 ± 0.75	52.18
	500	–0.412 ± 0.009	1.42 ± 0.24	68.70
	1000	–0.476 ± 0.011	6.97± 0.41	26.68

a Units: *E*_corr_ (V), *I*_corr_ (μA/cm²), η (%), *C* (concentration of inhibitor); legend for all PILs: A (2-HDEAF), PIL B (2-HDEAP), and PIL C (2-HDEAPe).

The cathode branch is next to the blank curve, and the anode branch is below, which marks the difference in current density (I) when the potential is plotted about the electrode area. This reduction in current density is characterized by forming a protective layer of the inhibitor on the metal surface, which degrades mainly in the anodic region over time and with the variation of current density. This protection can be observed in the morphological characterization results, where the metal surface with (PILs) presents a considerable reduction in degradation. In Table 3, the elements evaluated are *E*_corr_, corrosion potential, and *I*_corr_: current density to achieve the values of ηE: efficiency through the polarization technique. In sum, the increase in concentration favored the formation of a barrier layer on the mild steel surface by the inhibitor molecules, decreasing the electrochemical reaction rates.^85,90,91^

#### Electrochemical Impedance Spectroscopy (EIS) Measurements

Carbon steel corrosion conductance in saline solutions with and without PILs was investigated by electrochemical impedance spectroscopy (EIS) at 25 °C (Figure 4 and Table 4).

**Figure 4:**
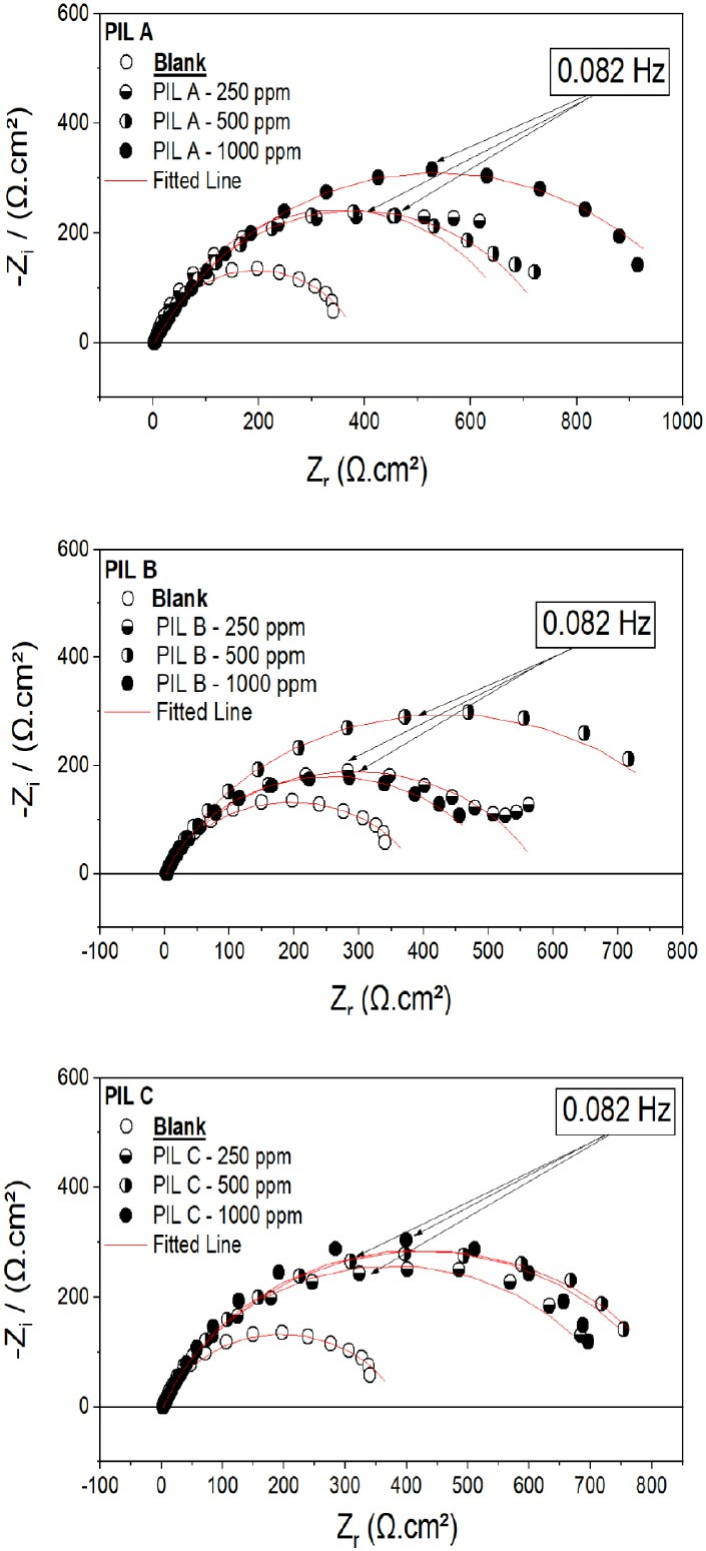
Impedance spectra of carbon steel (A36) in 3.5wt % NaCl in the presence and absence of PILs A, B, and C with different concentrations at open circuit potential.

**Table 4:** Impedance Factors of A36 in 3.5wt % NaCl with and without Different NaCl Concentrations

		CPE				
PILs (ppm)	Rs (Ω·cm^2^)	Y_0_(s^n^. Ω^–1^.cm^–2^) 0.10^–3^	n. 10^–2^	*C*_dl_ (F·cm^–2^) × 10^–3^	Rp (Ω·cm^2^)	IE (%)
Blank	1.78 ± 0.03	3.56 ± 1.23	75 ± 2	4.36 ± 2.15	432± 19	-
A-250	3.90 ± 0.46	8.75 ± 6.60	75 ± 3	2.85 ± 16.5	906 ± 78	51.50 ± 5.04
A-500	3.95 ± 0.24	1.89 ± 0.24	73 ± 1	2.43 ± 0.48	1038± 95	57.86 ± 4.09
A-1000	4.29 ± 0.86	1.27 ± 0.32	70 ± 3	1.73 ± 0.65	1463 ± 94	70.31 ± 1.83
B-250	2.44 ± 0.09	2.40 ± 1.92	74 ± 1	3.72 ± 3.10	932 ± 62	53.36 ± 2.99
B-500	3.73 ± 0.12	3.03 ± 0.30	76 ± 2	4.77 ± 1.79	1272 ± 65	65.88 ± 1.78
B-1000	2.79 ± 0.30	1.98 ± 0.32	73 ± 4	2.39 ± 0.39	852± 62	48.93 ± 3.91
C-250	3.07 ± 0.48	3.95 ± 1.49	75 ± 1	6.10 ± 3.13	831 ± 28	47.89 ± 1.82
C-500	3.85 ± 0.24	2.88 ± 0.42	75 ± 2	4.23 ± 0.81	1111 ± 28	61.05 ± 1.05
C-1000	3.18 ± 0.12	4.69 ± 0.52	76 ± 1	6.81 ± 0.89	700 ± 23	38.04 ± 2.00

Figure 4 presents the Nyquist plots for ASTM A36 carbon steel in 3.5 wt % NaCl solution, both with and without corrosion inhibitors. The results show that the semicircles observed in the high-frequency (HF) region are incomplete, which may be attributed to surface heterogeneity and the adsorption of chemical species onto the metal surface. Additionally, the reaction mechanism appears to be governed by charge transfer resistance, which is susceptible to corrosion by the electrolyte. The literature on corrosion inhibitors indicates that factors such as impurities, surface roughness, dislocations, and the adsorption of inhibitors on the material contribute to surface heterogeneity. These aspects can significantly influence the electrochemical behavior of the carbon steel in corrosive environments.^78,92–94^

The diameters of the capacitive loops gradually increased with the addition of small concentrations of PILs, without altering the overall shape within the studied concentration range (eqs 7 and 8). This behavior indicates the inhibitory effect of the protic ionic liquids. In particular, eqs 2 and 3 are used to calculate the inhibition efficiency and double-layer capacitance (*C*_dl_), respectively. Rp and Rp_inh_ are the polarization resistances in the presence and absence of inhibitors.^92,95^

7 $$\mathrm{I.E}\left(\%\right)=\frac{\left(\mathrm{Rp}\left(\mathrm{inh}\right)-\mathrm{Rp}\right)}{(\mathrm{Rp}\left(\mathrm{inh}\right)}x100$$

8 $$Cdl\left(\mathrm{F.}{\mathrm{cm}}^{-2}\right)={(QxR_{P}^{1-n})}^{1/n}$$

As pointed in Table 4, the final polarization resistance (*R*_p_) values for PIL A remained consistently high across the range of tested concentrations, exceeding 1,000 Ω·cm^2^. In this regard, this result suggests that PIL A provides more effective protection, regardless of its concentration. In contrast, for PILs B and C, an increase in concentration from 500 to 1000 ppm led to a noticeable reduction in the diameter of the capacitive loops. This decline is likely attributed to the saturation of the electrochemical double layer, which may have reached its maximum surface coverage, thereby limiting further inhibitor adsorption—an effect consistent with findings reported in the literature^50,96^

Overall, the impedance spectroscopy results demonstrate a clear enhancement in polarization resistance with the addition of protic ionic liquids, as evidenced by the enlargement of the capacitive loops compared to the blank sample. This indicates a strengthening of the protective barrier formed by the inhibitors. Moreover, the data presented in Figure 4 corroborate the findings from the PP curves, reinforcing the observed trends.

Among the three PILs tested, PIL A exhibited the most pronounced inhibitory effect, outperforming both PIL B and PIL C in terms of corrosion resistance.^96,97^ Thus, the impedance results indicate a visible increase in the intensity of the polarization resistance (Rp) caused by adding the PILs. This information was obtained by increasing the capacitive arcs compared to the blank value. Furthermore, the results shown in Figure 4 agree with what was observed in the potentiodynamic polarization. In sum, PIL A exhibited a more pronounced inhibitory effect compared to the other PILs, B and C.

In detail, a close inspection and discussion of the recorded, obtained, and calculated EIS data in Table 4 indicates that Rs values of ASTM A36 increase with the addition of the PILs molecules. The solution resistance (Rs) result is observed at low concentrations as low as 250 ppm (3.90 Ω·cm^2^) relative to the empty counterpart (1.78 Ω·cm^2^) and subsequently increases until the maximum value (4.29 Ω·cm^2^) is reached at 1000 ppm.

This is reflected in the adsorption process of PILs on the surface. They formed a stable protective layer that protected the surface of the A36 steel from attack by the NaCl electrolyte, thus reducing the corrosion rate of the steel. Moreover, the EIS results show that the *C*_dl_ values decrease with increasing Rs, and the decrease in the *C*_dl_ values is related to the replacement of water and aggressive ions with a high dielectric constant (ε) by absorbed molecules with low ε. Moreover, an increase in the thickness of the shielding protective film (T) over the A36 steel surface was observed, according to the Helmholtz model.

#### Critical Micellar Concentration (CMC)

The evaluation of the compounds at concentrations near or above their critical micelle concentration (CMC) was conducted to provide a comprehensive assessment of their corrosion inhibition behavior across different concentration regimes. Although it is well-established that micelle formation can influence adsorption processes and, consequently, the inhibition mechanism, investigating the performance both below and above the CMC is essential for understanding the full operational potential of these compounds.

In some cases, protective effects have been observed even in micellar regions, likely due to enhanced surface coverage or the formation of inhibitor aggregates that interact with the metal surface. Moreover, the choice to include concentrations above the CMC reflects realistic scenarios, as industrial applications often employ inhibitor concentrations that approach or exceed this threshold. The discussion of the results considers these aspects, emphasizing the relationship between concentration, micelle formation, and inhibition efficiency.

The critical micelle concentration (CMC) is a specific value at which the concentration of a particular surfactant in the solution changes the initial state of molecular solvation. However, most of the physical and chemical properties of the chemical system changed noticeably at this specific concentration. This effect is fundamental for theoretical and practical applications, including a better understanding of corrosion inhibitors and the maximum values of inhibitors in electrolytic systems.^98,99^

Figure 5 illustrates two different scenarios. First, PIL A has a much later stabilizing moment (after 620 ppm) than PIL B and C. This factor is likely related to a significant difference in the size of the carbon chain. Since formic acid is the smallest compound of the ionic liquid structure investigated in this study, a larger amount of this compound is needed to saturate the interaction with the surface of the metal. The relatively higher CMC of PIL A may explain why the best concentration of this inhibitor was obtained at 1000 ppm, considering that above 600 ppm, the electrolytic system was already saturated by the miscella formation.

**Figure 5:**
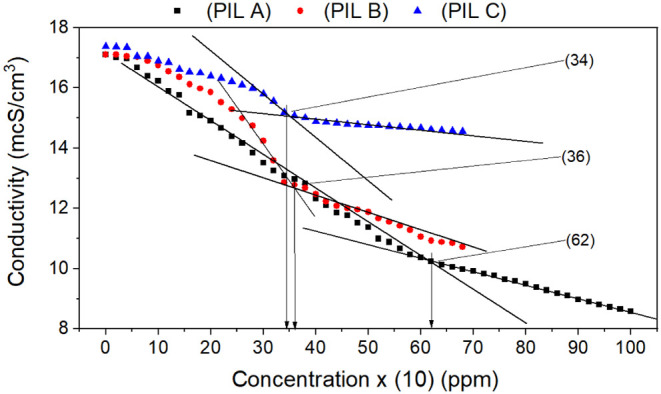
Variation in conductivity with inhibitor concentration for the carbon steel electrode in 3.5wt % NaCl solution at 25 °C.

The protic ionic liquids (B and C) exhibited a decrease in corrosion control efficiency at the highest concentration tested. This trend can be attributed to the system’s saturation, occurring at concentrations below 400 ppm (340 ppm for PIL C and 360 ppm for PIL B). Such findings are essential for elucidating the reaction mechanism, as the size of the molecules significantly affects the effectiveness of corrosion inhibition. Understanding this relationship helps clarify the limitations of these ionic liquids in corrosion control applications.

In his analysis, Funchs-Godec emphasizes that the critical micelle concentration (CMC) is influenced by various factors related to the compound and the solution (electrolyte), particularly the aqueous/saline environment. A distinctive significant factor is the ionic strength of the liquid, which affects the CMC value compared to its original value in pure water. Additionally, Funchs-Godec observed stability in the CMC curve during his investigation of quaternary ammonium salts as corrosion inhibitors on the surface of carbon steel in 2 M H_2_SO_4_. This research underscores the importance of environmental conditions in determining the effectiveness of these compounds.^99^

#### Surface Analysis

The preliminary topic of surface assessment is essential for establishing the visual and qualitative context of the corrosion inhibition performance observed in subsequent electrochemical analyses. Morphological evaluation through microscopy techniques serves as a preliminary but crucial step in identifying surface changes associated with corrosion processes and inhibitor action. In this study, surface imaging was employed to assess the macroscopic and microscopic features of the carbon steel samples before and after exposure to the electrolyte containing corrosion inhibitors (CI).

This approach enables the identification of phenomena such as excessive oxide layer formation, changes in surface roughness, and visible degradation, which are indicative of the inhibitor’s protective capacity.

A visual inspection can provide insights into the physicochemical interactions between the inhibitor and the corrosive medium, including observations related to solubility, dispersion, and changes in the solution’s appearance, such as color variations which may suggest compound instability, reaction byproducts, or micelle formation at higher concentrations. These morphological findings support the interpretation of electrochemical data by offering evidence of inhibitor performance at the surface level, thereby validating trends in polarization resistance or corrosion current. Thus, surface analysis contributes not only to the qualitative evaluation of inhibition effectiveness but also complements and reinforces the electrochemical investigation presented in this work. Morphological evaluation is a visual and qualitative method used to assess the effectiveness of corrosion inhibition before and after electrochemical testing. In this study, microscopy techniques were employed to provide an initial overview of the material’s surface condition.^37^ This micrograph includes information about the compounds evaluated in the experiments with CI, such as whether there was excessive oxide production, a change in solution color, and the solubility of the inhibitor in the electrolyte.

#### Scanning Electron Microscopy Analysis

Scanning electron microscopy (SEM) assessed the material’s morphology after polarization tests with 1000 ppm protic ionic liquids (PIL A-C). The ASTM A36 steel was exposed to 3.5 wt % NaCl electrolyte solution with and without (b) PIL A (2-HDEAF), (c) PIL B (2-HDEAP), and (d) PIL C (2-HDEAPe) after 1-h open circuit potential (OCP) and potentiodynamic polarization tests at room temperature (25 °C). The surface evaluation of the metal specimens, both in the absence and presence of corrosion inhibitors, was conducted using scanning electron microscopy (SEM) at a concentration of 1000 ppm.

This concentration was selected as it represented the condition under which the highest corrosion protection efficiency was observed in the CI evaluation. Agreed the limited availability of the SEM equipment and the high demand for its use, it was not feasible to perform surface characterization for all tested concentrations. Therefore, the decision to focus on the optimized condition was made to ensure a meaningful and representative morphological analysis of the inhibitor’s performance. This approach allows for a clearer understanding of the protective mechanisms at play, particularly in terms of surface coverage, oxide formation, and the extent of corrosion attack, under the most effective inhibitor concentration in a 3.5% NaCl saline medium. Carbon steel in saline environment without the inhibitor (Figure 6, Section A) exhibited an irregular and damaged surface, indicating that the outer region of the studied material was drastically damaged by exposure to salty and acidic environments, as described in the literature on corrosion inhibitors.^100^ It was found that the damage to the studied part was significantly reduced, and the surface had a uniform appearance without significant damage owing to the corrosion process.

**Figure 6:**
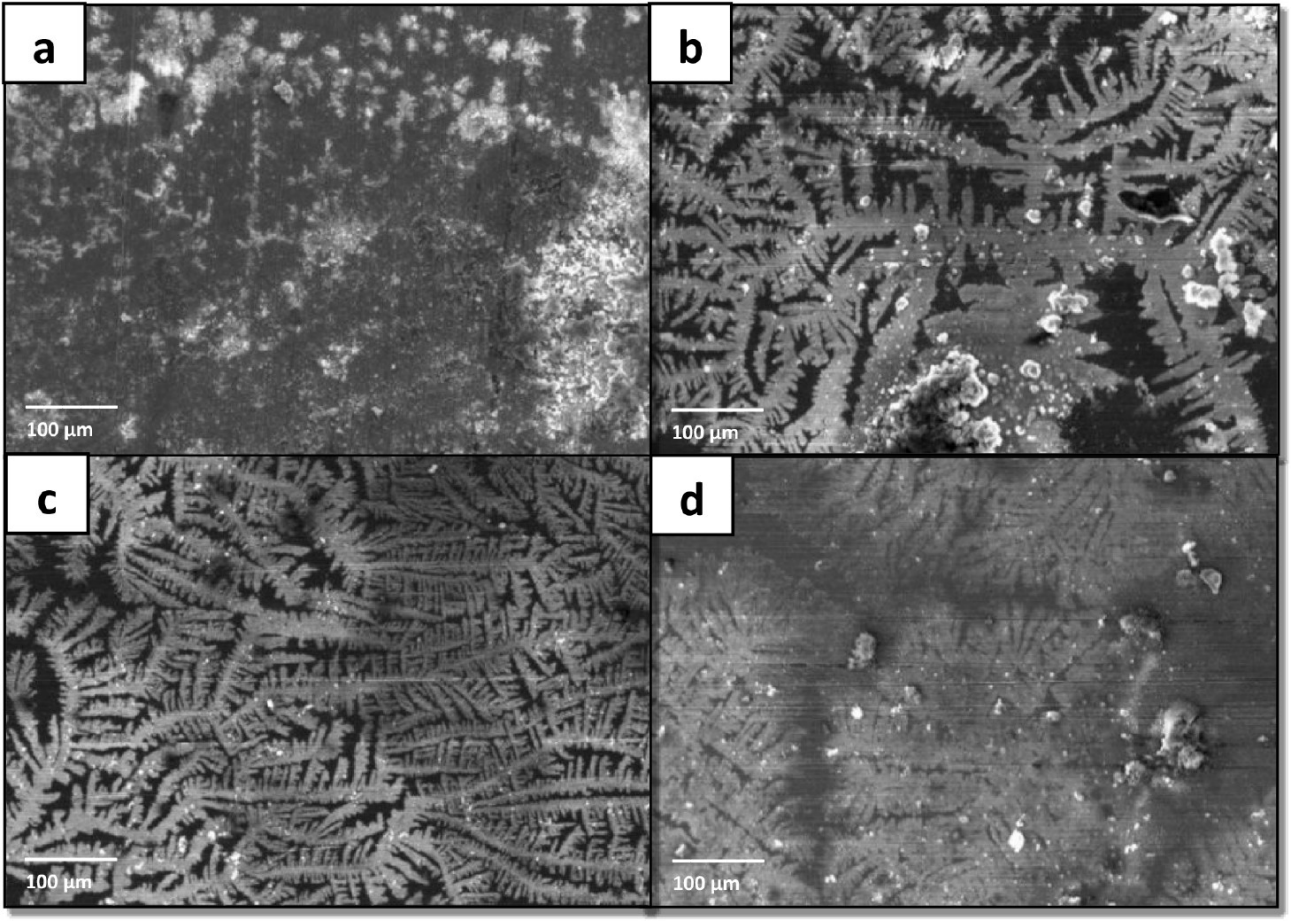
SEM micrographs (1000×) of mild carbon steel: (a) absence of PILs and in the presence of 1000 ppm of (b) PIL A (2-HDEAF), (c) PIL B (2-HDEAP), and (d) PIL C (2-HDEAPe) after polarization tests at 25 °C.

It was observed in the presence of all the PILs studied (Figure 6—B until D). Several corrosion products accumulated on these surfaces, which is the primary method for evaluating the steel. In detail, certain areas without corrosive effects are also present, confirming the material’s protection due to the adsorption of CI.^50^ These results assists to confirm the effectiveness of the PILs as CI on ASTM carbon steel in a saline medium and corroborate the results of the electrochemical tests. The steel samples were immersed in a 3.5 wt % saline solution containing PILs A, B, and C (Figure 6), resulting in visibly smoother surfaces after the polarization tests. This observation indicates that the material received effective chemical protection against corrosion from the protic ionic liquids. The protective effect observed through morphological analysis is consistent with the results obtained from weight loss measurements and electrochemical tests. These findings highlight the value of morphological evaluation as a complementary and rapid method for confirming the effectiveness of corrosion inhibitors. Such techniques offer a visual and accessible means of validating the performance of inhibitors, thereby enhancing the reliability of electrolyte-based assessments.

This comprehensive evaluation of the metallic surface is frequently reported in corrosion literature. For instance, Santana Rodríguez et al. employed X-ray diffraction (XRD) and scanning electron microscopy (SEM) to identify and characterize the various corrosion products formed corrosion conditions.

The formation of corrosion products on metallic samples exhibited notable variation depending on the medium applied. Significant material degradation and rapid oxide formation were observed in saline environments that simulate seawater conditions, primarily due to the interaction of chloride ions with the metal surface.^101^ This comprehensive assessment of metallic surfaces is commonly found in corrosion literature.

For instance, Santana Rodríguez et al. utilized X-ray diffraction (XRD) and scanning electron microscopy (SEM) to identify and characterize the corrosion products formed in various urban, marine, and marine-industrial settings. Notable variations in corrosion product formation were observed depending on the medium applied. In saline environments, simulating seawater conditions, significant material degradation and rapid oxide production occurred due to chloride ion interactions with the metal surface. XRD analysis was pivotal in examining the oxides formed, providing insights into the interactions between inhibitor molecules and the metal substrate, which is crucial for developing effective corrosion mitigation strategies.

#### Atomic Force Microscopy (AFM)

Three-dimensional atomic force microscopy (AFM) micrographs were obtained for ASTM A36 carbon steel samples in the absence and presence of the chosen corrosion inhibitor compound, PIL A. The electrochemical and gravimetric results presented in Section 3.1 indicated that PIL A exhibited the most favorable performance, justifying its selection for the AFM investigation. In the absence of the chosen inhibitor in the electrolytic medium, the surface of carbon steel experienced severe damage within 24 h immersion because of the highly aggressive nature, which was potentiated by the action of chloride ions present in the solution.

In detail, the phase images (Figure 7) provide an overview of the material’s surface evaluated through the meticulous technique of Atomic Force Microscopy (AFM). In these results, it is possible to estimate the average roughness profile. Data considered crucial for the investigation of corrosion inhibitors, where it is possible to evaluate in a nanometric way how the surface of the material is after the period in electrolytic (NaCl 3.5 wt % at a temperature of 25 °C) with and without the presence of the inhibitor.

**Figure 7:**
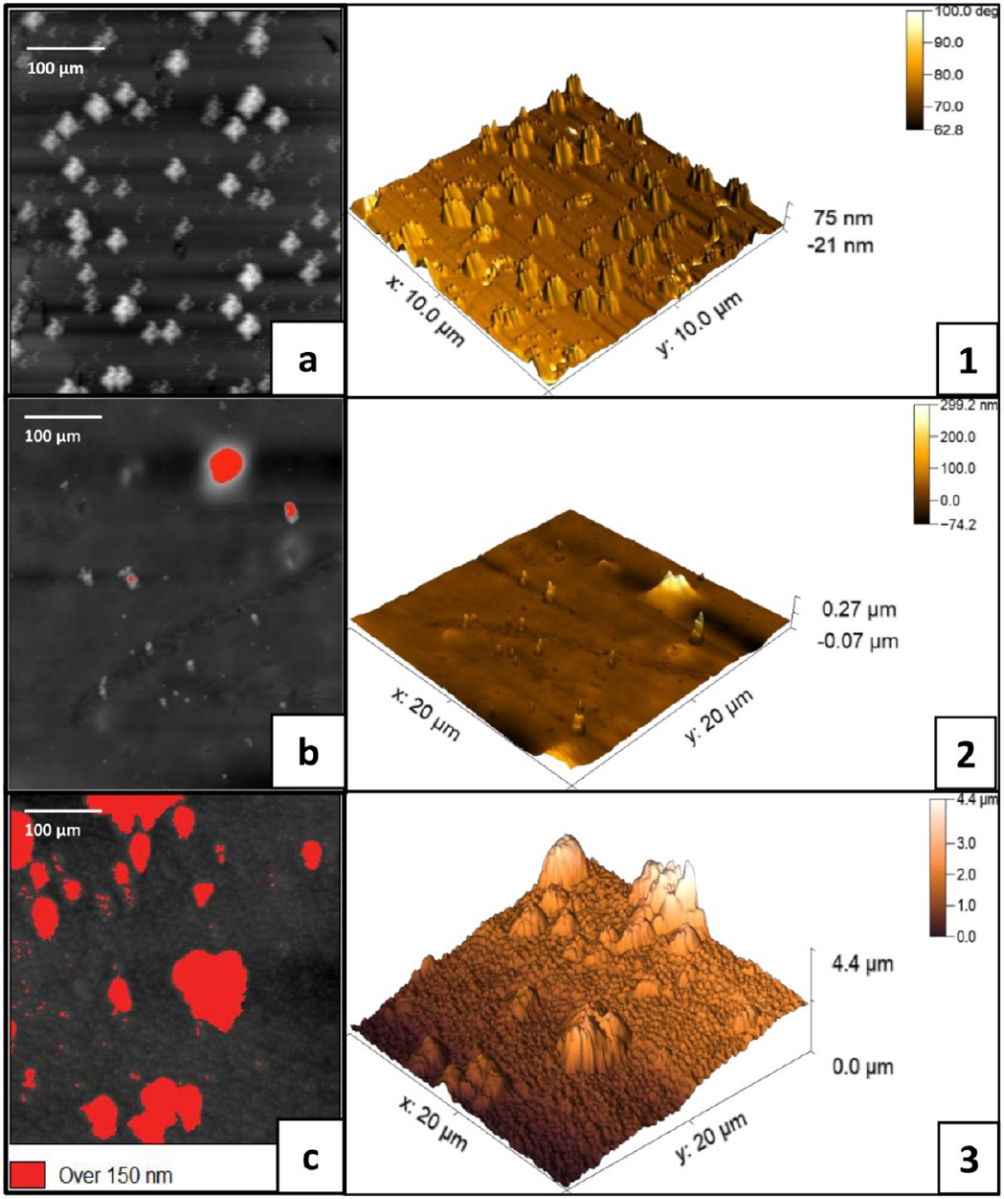
AFM phase (left) and 3D topography (right) images of A36 carbon steel after immersion in 3.5 wt % NaCl with the best condition corrosion inhibitors: (1, a) PIL A (2-HDEAF, 1000 ppm), (2, b) polished control, and (3, c) blank NaCl solution.

Generally, AFM results confirmed a correlation between the remission of roughness and the addition of a corrosion inhibitor in an electrolytic system. Still, on the AFM technique, it was decided to use a parallel view to evaluate the 3D profile (Figure 7, Sections 1–3). This evaluation highlights specific regions in the result using a threshold marked with a fixed height of 150 nm. The areas in the 3D profile that exceed this value would be highlighted with a distinct color, in the red case, exceeding the fixed size, confirming its high deterioration in parallel with the numerical value of the average roughness (Figure 7 Sections A–C).

Evaluating (Figure 7), the roughness and thickness of the exterior have noticeably increased. This observation reflects the film layer over the metal surface, possibly through a physisorption process, due to the chemical structure of the protic ionic liquids. These findings confirm the previously presented results in the adsorption isotherm section. It is assumed that this layer effectively covers the entire surface area of the material, aided by the corrosion inhibitor (PIL A) at an optimized concentration of 1000 ppm. The measured range of roughness for the treated surface is between 0.27 and −0.07 μm. This contrasts with the untreated surface exposed to a saline environment, which exhibits peaks of corrosion products reaching heights close to 4.4 μm. In comparison, the average surface roughness (ASR) measured for the uninhibited samples exposed to 3.5 wt % NaCl for 24 h was 185 nm (Figure 7, Section C). However, in the presence of PIL A at its optimal concentration (1000 ppm), the A.S.R. was significantly reduced to 11.8 nm (Figure 7, Section A), a value close to that of the control surface (8 nm), which had not been exposed to the corrosive medium (Figure 7, Section B). These considerably lower roughness values indicate that the adsorption of PIL A onto the steel surface provides effective protection against corrosion in a saline environment. Figure 8 presents two-dimensional contour maps corresponding to the uninhibited sample, the sample treated with 1000 ppm PIL A, and the polished control surface, respectively. The addition of PIL A led to a marked reduction in surface roughness, confirming its inhibitory action. Comparative analysis highlights the significant difference among the samples and supports the formation of a protective inhibitor film on the metal surface.

**Figure 8:**
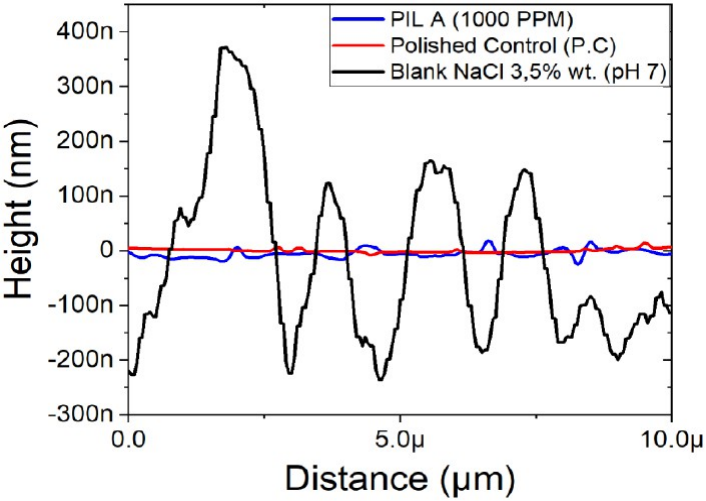
2D roughness profile of AFM images: 1) PIL A (1000 ppm), 2) Polished Control, and 3) Blank NaCl 3.5wt %.

Finally, the literature that discusses the relationship between the protective capacity of a corrosion inhibitor and its average roughness values indicates a baseline increase of up to 100 nm in the average surface roughness of the material after the addition of these protective compounds in solution, compared to a blank measuring up to 1.5 μm.^13,60,102,103^ Thus, the corrosion inhibitors that resulted in an average surface roughness of 11 nm very close to that of the polished control surface (8 nm) demonstrate high efficiency in forming a protective film, significantly minimizing surface degradation.

#### Characterization of Corrosion Products by XRD (X-ray Diffraction)

This type of comprehensive evaluation of metallic surfaces is well established in the corrosion literature. For example, Santana Rodríguez et al. used X-ray diffraction (XRD) and scanning electron microscopy (SEM) to identify and characterize corrosion products formed under different environmental conditions, including urban, marine, and marine-industrial atmospheres. Their findings revealed significant variations in the nature and extent of corrosion products depending on the exposure environment. In particular, saline conditions simulating seawater led to pronounced material degradation and rapid oxide formation due to the aggressive interaction of chloride ions with the metal surface. XRD analysis was instrumental in identifying the oxides present and clarifying the interactions between inhibitor molecules and the metal substrate insights that are essential for the development of effective corrosion mitigation strategies.

There is a notable difference in the phases found in the diffraction patterns of the blank sample without CI compared to the protic-ionic liquids (inhibitors) (Figure 9). The main difference observed in the diffraction study with the addition of protic ionic liquids in saline solution is the presence of specific phases of corrosion products that serve as an indication of oxide formation, especially Goethite. Initially, halite phases were present, resulting from the interaction between Na^+^ and Cl^–^ ions in the saline electrolyte and the steel surface (A36). The occurrence of this phase (halite) was understandable because of the high concentration of the solution (3.5 wt %) used in the immersion tests during a long period (30 days).

**Figure 9:**
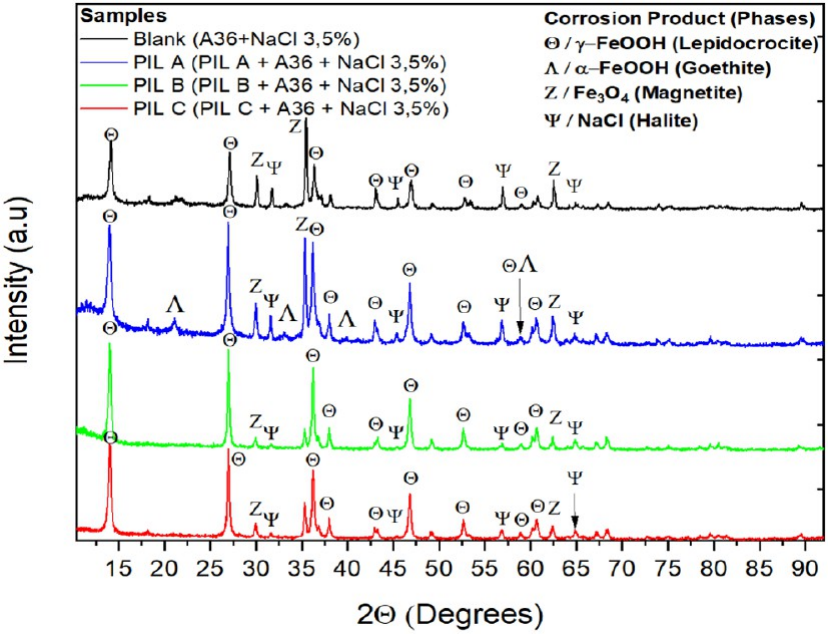
X-ray diffraction (XRD) patterns of rust products in the absence (blank) and presence of 1000 ppm of three different protic ionic liquids (PIL A-C).

In corrosion studies involving aqueous media, two key conditions are typically considered: exposure to air and immersion in aqueous solutions. This study employed an open system to simulate an aerated environment, aiming to better understand the corrosion inhibition mechanisms. X-ray diffraction was used as a critical technique for identifying oxides and hydroxides formed on carbon steel in oxygen-rich environments. The main corrosion products identified were lepidocrocite, magnetite, goethite, and halite. Magnetite, lepidocrocite, and halite were consistently detected in all samples, both with and without corrosion inhibitors (Table 5).

**Table 5:** X-Ray Patterns of Rust Product Phases (Oxides and Hydroxides)^a^

Samples	Mineral	Oxide	Hydroxide	
Blank	Halite (NaCl)	Magnetite (Fe_3_O_4_)	Lepidocrocite (γ-FeOOH)	-
A	Halite (NaCl)	Magnetite (Fe_3_O_4_)	Lepidocrocite (γ-FeOOH)	Goethite (α- FeOOH)
B	Halite (NaCl)	Magnetite (Fe_3_O_4_)	Lepidocrocite (γ-FeOOH)	-
C	Halite (NaCl)	Magnetite (Fe_3_O_4_)	Lepidocrocite (γ-FeOOH)	-

a A: PIL-A; B: PIL B; C: PIL C.

The analysis of corrosion products provides valuable insights into the environmental conditions, as the formation of particular oxide phases reflects the characteristics of the surrounding medium. This approach offers a robust charter for understanding the behavior of electrolytic systems under aerobic conditions. The formation of specific crystalline phases during prolonged exposure in the presence of corrosion inhibitors helps elucidate the interactions between the steel surface and the inhibitors.^61^

An internal layer is formed by goethite, which has an amorphous structure with a stable, compact, and denser structure. Faced with these characteristics, the presence of this phase on the metallic surface brings a protective character to the metallic surface, according to the literature about atmospheric corrosion.^104^ It is worth mentioning that the study of atmospheric corrosion considers long periods.

A factor that can be considered is that if there are specific phases, the protective capacity of the material can be enhanced, either with an inhibitor or an anticorrosion coating.^105^ The techniques applied (electrochemistry, weight loss, and microscopy) proved that PIL-A presented the best efficiency against corrosion resistance.

The presence of each phase in the blank sample was also observed in the PIL-A spectra (Figure 9). However, the significant difference was the presence of a goethite phase in the PIL-A spectra. This observation suggests that the superior protective performance of PIL-A as a corrosion inhibitor may be attributed to the formation of a denser and more compact layer on A36 carbon steel after 30 days of immersion, resulting in inhibition efficiencies exceeding 85%.

The presence of oxide and hydroxide phases such as lepidocrocite, hematite, and magnetite is typically observed above the goethite layer. Furthermore, the formation of lepidocrocite and magnetite within the outer region of goethite on metallic surfaces is commonly associated with exposure to saline atmospheric conditions.^104^

This layer of phases has adverse characteristics due to the high porosity of the material and the permeation of contaminants O^2^, Cl^–^, and Na^+^ from the environment, catalyzing the corrosive process and promoting a faster degradation of the material for a long time. However, in systems with the formation of more internal and dense layers, such as goethite, some authors argue that there is a protective factor for its presence in steel samples, particularly carbon and stainless steel.

This layer would provide a substantial barrier over the steel to protect against contaminant permeation, thereby maintaining its integrity. Interestingly, this phase was present in the XDR spectrum of the best corrosion inhibitor tested in this study (PIL-A).^106^ Still, regarding the phases present in the XDR, according to Fuente (2011 ^107^), magnetite is usually associated with forming a corrosion layer of low protection over the material (steel).

Most authors have reinforced goethite and ferrihydrite’s protective functions over metallic surfaces; however, only the goethite phase was observed in experiments with protonic ionic liquids (PILs). On the other hand, hematite and magnetite have nonprotective capacities, which are justified by their high porosity and instability over the steel surface.

Finally, two distinct situations were observed for PIL B and C. Both had the same phases as the blank sample (without PILs). According to the literature on morphological studies, the presence of certain phases of iron oxides depends on the pH of the electrolytic medium. It may evolve into a final stage, such as goethite.

For example, ferrihydrite, a transient phase under aqueous conditions, gradually transforms into a more stable and thermodynamically favorable crystalline phase typically goethite or hematite depending on the pH and temperature of the reaction medium.^108^ Usually, this structural modification occurs under specific circumstances, such as temperature (27–70 °C) and pH (7.5–9.0).^109^ Indeed, a plausible justification exists for developing several phases in the metallic area, specifically goethite and ferrihydrite.^110,111^ During the morphological evaluation of the steel surface studied, the pH and conductivity values were observed by adding all PILs in the NaCl solution (3.5wt %) for 24, 48, and 72 h. It was found that the pH value in the addition of PIL A was initially from 8.4 to 6.7 with a period of 72 h; according to the stability range, this arrangement with a slightly neutral pH allows the successful formation of the XRD-proven goethite phase (information in the Supporting Information).

The corrosion inhibitor PIL C demonstrated intermediate but satisfactory performance. No distinct crystalline phases associated with PIL B were observed, with the primary structural difference between the two PILs being the length of the carbon chain. The longer carbon chain in PIL C resulted in improved surface coverage, thereby enhancing its protective capacity compared to PIL B. In contrast, PIL B exhibited the least effective corrosion inhibition among the PILs evaluated. Its lower performance is attributed to its higher water solubility and reduced surface coverage, a consequence of its shorter carbon chain. This limited protective ability was further supported by the XRD analysis (Figure 9), which revealed phase compositions similar to those observed in the uninhibited system. In summary, while PILs B and C provided comparable protection, the difference in performance is clearly linked to the length of their alkyl chains: PIL B is derived from propanoic acid (three carbon atoms), whereas PIL C is based on pentanoic acid (five carbon atoms).

#### Scanning Electron Microscopy (SEM) Assessment for Corrosion Product Identification

The diversity of shapes and sizes obtained from identification with the assistance of a scanning electron microscope (SEM) after 24 h (saline solution 3.5 wt %) can be seen in Figures 10 and 11 with distinct individualities, such as for the blank, without inhibitors (PIL A), the clear presence of significant phases on the metallic surface such as lepidocrocite (γ-FeOOH) and magnetite (Fe_3_O_4_) is emphasized, dominantly with different shapes and sizes (details in Table 6). It is important to note that high porosity significantly compromises the integrity of the oxide/oxyhydroxide layer. The evaluation of PILs 1 and 2 revealed the presence of distinct forms of goethite (α-FeOOH) on the surface, contributing to the formation of a compact and dense corrosion product layer.

**Table 6:** Corrosion Produces Information about Morphology Based on Literature

		Forms			
Iron oxide/oxyhydroxide	Colors	Ref 113	Ref 114	Refs 115 and 116	Ref 117
Goethite (α-FeOOH)	Yellowish to reddish to dark brown or black	Acicular	Star, hexagons, bipyramids, cubes	Cloud, thin, flat cotton-balls	Needle, shaped, laths whiskers
Lepidocrocite (γ-FeOOH)	Black to Dark Gray	Laths	Tablets, plates, diamonds, cubes	Thick plates, sandy, thick sheet	Laminar, sandy grain, worm nest, bird’s nest
Magnetite (Fe_3_O_4_)	Strong red to red close to orange	Octahedra	Octahedral, rhombic dodecahedra	Flat layer, grain	Blackish circular rings

**Figure 10:**
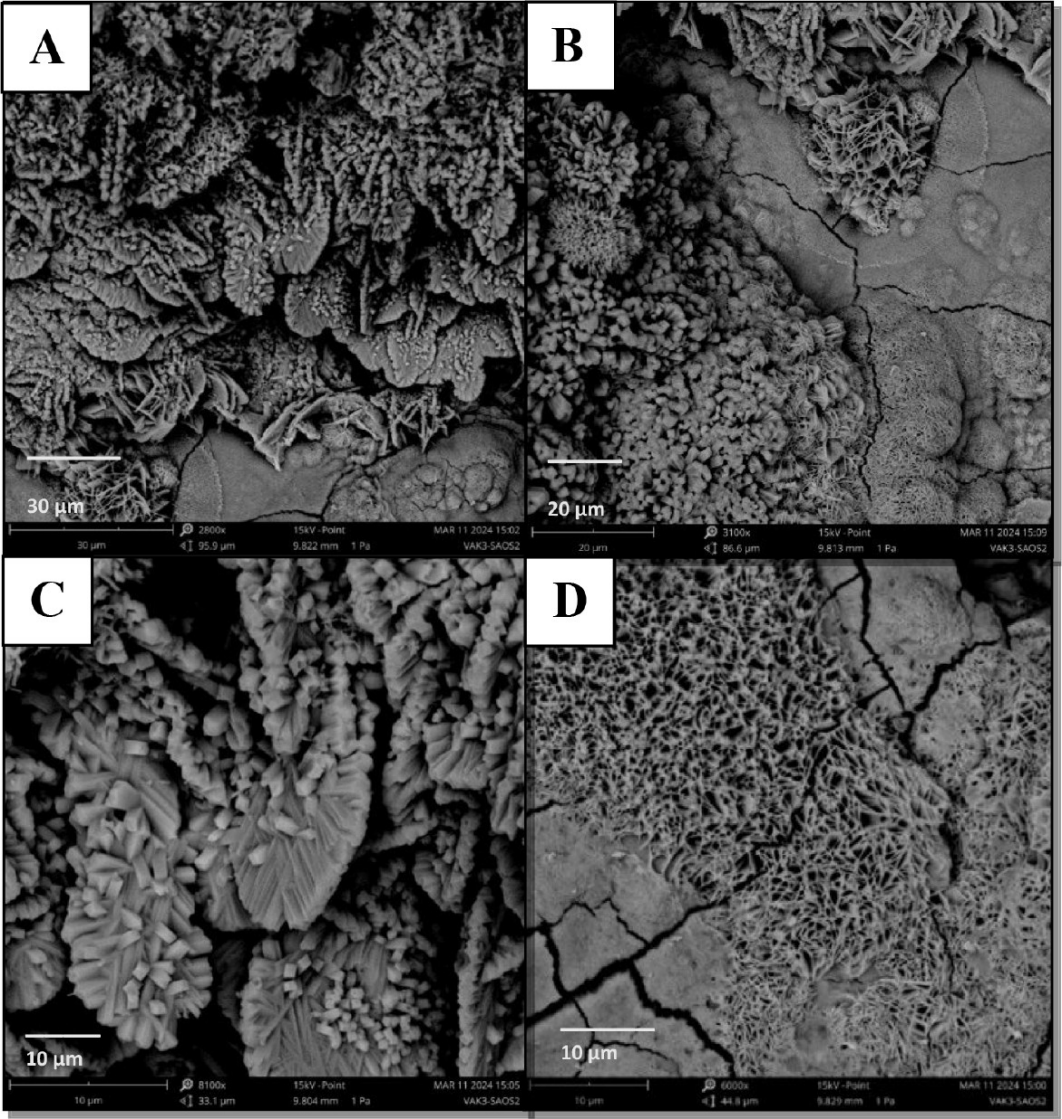
Blank sample carbon steel immersion in NaCl solution 3.5 wt % for 24 h A—Region 1 (30 μm), B—Region 2 (20 μm), C—Region 310 μm), D—Region 4 (10 μm).

**Figure 11:**
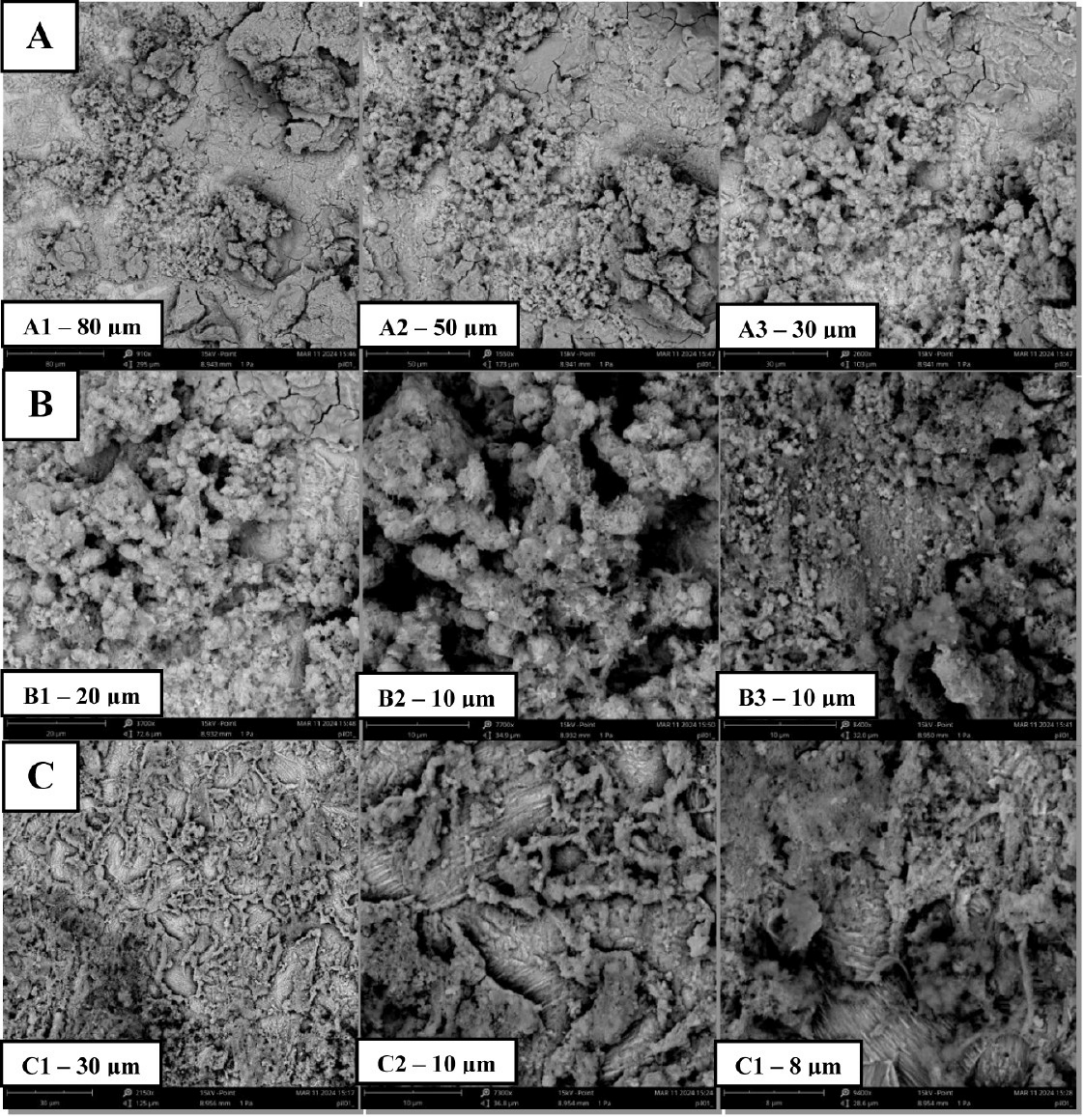
SEM micrographs of A36 carbon steel after 24 h immersion in 3.5 wt % NaCl in the presence of PIL A (2-HDEAF). Images (A–C) show three distinct regions of the same inhibited sample; subimages (A1–A3, B1–B3, and C1–C3) correspond to different locations within each region, illustrating local surface heterogeneity under identical inhibition conditions.

The characteristics of the observed corrosion products were assessed based on established criteria reported in the corrosion literature (Table 6).^43,112^

The image of the sample blank (Figure 10) reveals the deposit of corrosion products on the surface. In Figure 10 (sections A and B), a significant accumulation of magnetite (sand crystals) can be observed on the surface of the micrograph, arranged in agglomerates. Furthermore, in the micrograph, a characteristic formation of lepidocrocite oxyhydroxide can be observed in the shape of a worm’s nest, which is widely discussed in the literature on corrosion.^114^

In the final region of the micrograph (Sections C and D), an increased number of magnetite crystals, also known as sand crystals, can be observed just below the bird’s nest formations, creating a generalized agglomerate adjacent to flakes near a prominent crack. Literature reports that these formations (oxides/oxyhydroxides) exhibit high permeation rates when present on the surface, and such corrosion products tend to facilitate the permeation of specific ions.

Notably, chloride ions are particularly detrimental to the steel surface, as their permeation can lead to significant degradation in surface integrity. Thus, given this high permeation, the possibility of crack formation in the oxides/oxyhydroxides found on the surface is associated with the fact that these crystalline formations are not compact and facilitate the access of chloride ions to the interior of this layer.

In Figure 10 (Section B), detecting an accumulation similar to that reported in Section A is achievable. However, in this micrograph, it is possible to observe a “dintless” region of the sample without much oxide/oxyhydroxide agglomerate.

In this region, it is clear that the saline electrolyte causes a considerable accumulation of corrosion products on the surface, with porous areas and cracks. Again, the phases indicated the formation of magnetite with sand and geometric crystals. There is a bird’s nest, worm’s nest, plates, needles, and globule formation for lepidocrocite formation.

Moreover, as shown in Figure 10, in the micrographs of sections C and D, the primary purpose of the investigation was an intricate view of magnetite formation with crystalline encasements (section C) and the formation of lepidocrocite with the bird’s nest and the structures in the form of needles with entrances, thus allowing good permeation of these types of oxides/oxyhydroxides (section D).

In section D, it is noted that the crack is directly associated with this type of arrangement, resulting in the fragility of this structure and high porosity. Regarding Figure 11, to appropriately evaluate the oxides identified via scanning electron microscopy, each magnification was divided into A1 (80 μm), A2 (20 μm), and A3 (10 μm) for each region evaluated: A, B, and C. In Figure 11, the carbon steel surface was assessed after adding the two inhibitors, PIL A.

The differential phase intended to be identified through microscopy is goethite (α-FeOOH), typically manifesting as cotton balls, grassy tufts, laths, or needles. The material examined in Figure 11, Section A, initially exhibits the presence of goethite formations at the superior left and center of the image, appearing as a cloud structure.

Indeed, this formation is a preliminary stage in developing the goethite structure, also found with lepidocrocite plates (bird’s nest formation).

Moreover, the center of the micrograph displays a notable accumulation of goethite phases in an agglomerate form. This formation indicates the potential for cotton ball-like structures to agglomerate, resulting in an overlaid and dense complex capable of protecting the metal surface from the permeation of specific ions, such as chloride.

Figure 11, section A1, exhibits large cracks near the oxides (lepidocrocite and magnetite). The deposit of oxides, predominantly goethite (cotton ball and grassy type), forms agglomerate due to their rounded/circular morphology, primarily observed in the superior and central regions of the micrograph.

This phenomenon is frequently reported in the literature.^113–117^ Furthermore, the Lepidocrocite phase is present in the superior and lower areas of the micrograph, manifesting in formations resembling a worm’s nest and a bird’s nest, which are characteristics of this oxyhydroxide. Notably, the crack propagation observed in the oxide layer is directly associated with the lepidocrocite phase (worm nest). Besides, on the lepidocrocite phase, laminar phases form a structure analogous to a boundary in the superior and inferior regions on the left side of the micrograph.

Another oxide detected on the material’s surface through microscopy was magnetite, notably concentrated in the lower and central regions, where well-distributed, geometrically defined sand crystals were observed. Section A2 provides a closer view (20 μm) of oxyhydroxide formations on the carbon steel surface, revealing goethite formations of particular significance.

The goethite formations appear as spherical aggregates layered upon one another, creating an agglomerate characteristic of oxyhydroxides, a feature extensively documented in the literature. Section A3, shown at higher magnification (10 μm), provides a detailed view of the oxides (magnetite) and oxyhydroxides (lepidocrocite) formed on the surface of A36 carbon steel. Notably, magnetite crystals with a distinctive octahedral structure are observed clustering alongside other phases, such as goethite, resulting in a well-defined agglomeration. Additionally, characteristic “worm nest” structures were identified.

The oxyhydroxide phase, lepidocrocite (γ-FeOOH), is commonly described as a highly permeable region within the corrosion product layer. As illustrated in Figure 11, particularly in section B1, lepidocrocite is found in the upper layer as worm nests—a morphology typical of saline environments where elevated concentrations of aggressive chloride ions promote its formation during iron exposure.^114,117^

In the region under evaluation, a notably widely spaced area was observed, characterized by a significant presence of pores and several fissures. This observation confirms that the high degree of porosity plays an essential role in the process of corrosion and, consequently, in the formation of oxides on the material’s surface. The porous nature of the area creates conditions that facilitate the access and accumulation of corrosive agents, making it a key factor to consider when evaluating oxide development in general.

Figure 11 provides a detailed representation of this area, dividing it into three distinct regions for a more systematic analysis. Region 1 (labeled A) includes subregions A1, A2, and A3, with dimensions of 80 μm, 50 μm, and 30 μm, respectively. Region 2 (B) contains subregions B1, B2, and B3, measuring 20 μm, 10 μm, and 10 μm. Lastly, Region 3 (C) is composed of subregions C1, C2, and C3, with sizes of 30 μm, 10 μm, and 8 μm. This subdivision allows for a more focused investigation of localized morphologies and phases within the corrosion products.

Particularly, the analysis of subregion B2 reveals the significant presence of goethite (α-FeOOH), as illustrated in Figure 11. The characteristic cotton ball and cloudy morphologies observed in this area strongly support the identification of goethite on the metal surface. Additionally, other oxide and oxyhydroxide structures were identified, including worm nests, globular formations, lepidocrocite (γ-FeOOH), and sandy crystals linked to magnetite (Fe_3_O_4_). These structures began to form within the initial 24 h of exposure to the corrosive environment. Importantly, the agglomerate regions formed by the interaction of goethite and magnetite phases exhibited vacancies and cracks.

These structural openings serve as critical pathways that can significantly enhance the permeation of chloride ions during prolonged exposure, thereby influencing the corrosion process and oxide growth dynamics over time. In section B3, the focus was on obtaining higher magnification to more accurately observe the phases of goethite (cotton balls and clouds), magnetite (geometric crystals), and lepidocrocite (sand crystals).

In conclusion, the discussion of the results in Figure 11 culminates in section C. Specifically, in section C1, a distinct edge or barrier formed by the corrosion products on the surface of the carbon steel was observed. This protuberance is probably the result of the accumulation of laminar structures of lepidocrocite (dish-like formations), goethite (cotton ball-like formations), and magnetite (sandy crystals). These formations tend to create divisions among the oxides due to the accumulation of agglomerate. Additionally, within these divisions, it is possible to discern various formations.

For section C2, a more detailed sample evaluation is possible using the SEM technique with a magnification of 10 μm. In the center and at the edges of the microscopic images, the barriers formed by the agglomerate of many different phases have the same phase inside, namely the goethite lamellae. This formation is essential for creating a compact and dense layer of oxides/oxyhydroxides over the material to protect it from chlorine penetration from the salt solution.

Similarly, sandy crystals, cotton balls, and worm nests can be observed above this formation (goethite laths). In addition, it should be emphasized that in these images (Figure 11), referring to PILs A, no large cracks are visible, as observed in Figure 10 in the control without the addition of inhibitors, confirming that the formation of this dense and compact layer of oxides can protect the carbon steel from ion permeation. Finally, section C3 presents a highly detailed evaluation with a magnification of 8 μm. This reduction aims to assess the proximity of the identified oxides, surface cracks, and the system’s porosity (corrosion products). The image focuses on barrier formations by the previously described agglomerate and highlights lamellae and cotton balls (goethite), sand crystals, and worm nests (lepidocrocite).

#### Energy Dispersive X-ray (EDS) Mapping for Oxides and Oxyhydroxides Chemical Composition Evaluation

Initially, the elemental differences in the morphology of the carbon steel surface induced by the corrosion process in a saline environment can be evaluated/understood as a substantial indicator in understanding the inhibition mechanism of the compounds used to protect the material. Thus, when assessing the composition table of the evaluated elements and the resulting map, it was confirmed that the surfaces of the working electrodes (samples) presented homogeneity after the addition of the corrosion inhibitors (PILs).

There were also several regions with high concentrations of Na, Cl, O, and N on the surface, as confirmed by elemental mapping via Energy Dispersive Spectroscopy (EDS) (Figures 12 and 13). In essence, quantitative results of the surface compositions are given in Tables 6, 7, and 8. In this way, Table 7 displays that the elemental composition is different for the various surfaces compared with 7 and 8 because it is possible to associate the values with the permeability of elements and accumulations. In detail, the EDS displayed in Figures 12 to 13 show that the selected elements (O, Fe, Na, C, N, Cl) represented by the colors (blue, red, green, yellow, purple, and cyan) to emphasize the amount in % that was found in the EDS results after the immersion period (24 h).

**Table 7:** Elemental Composition of A36 Outside at the End of the 24-h Immersion Experiment for the Control Solution (NaCl wt. 3.5%) without Corrosion Inhibitors (PILs)

Element	Atomic Concentration	Weight Concentration
Fe	47.46	24.52
O	7.45	1.10
N	0.71	0.22
C	1.11	0.12
Cl	0.82	0.27
Na	0.23	0.05

**Figure 12:**
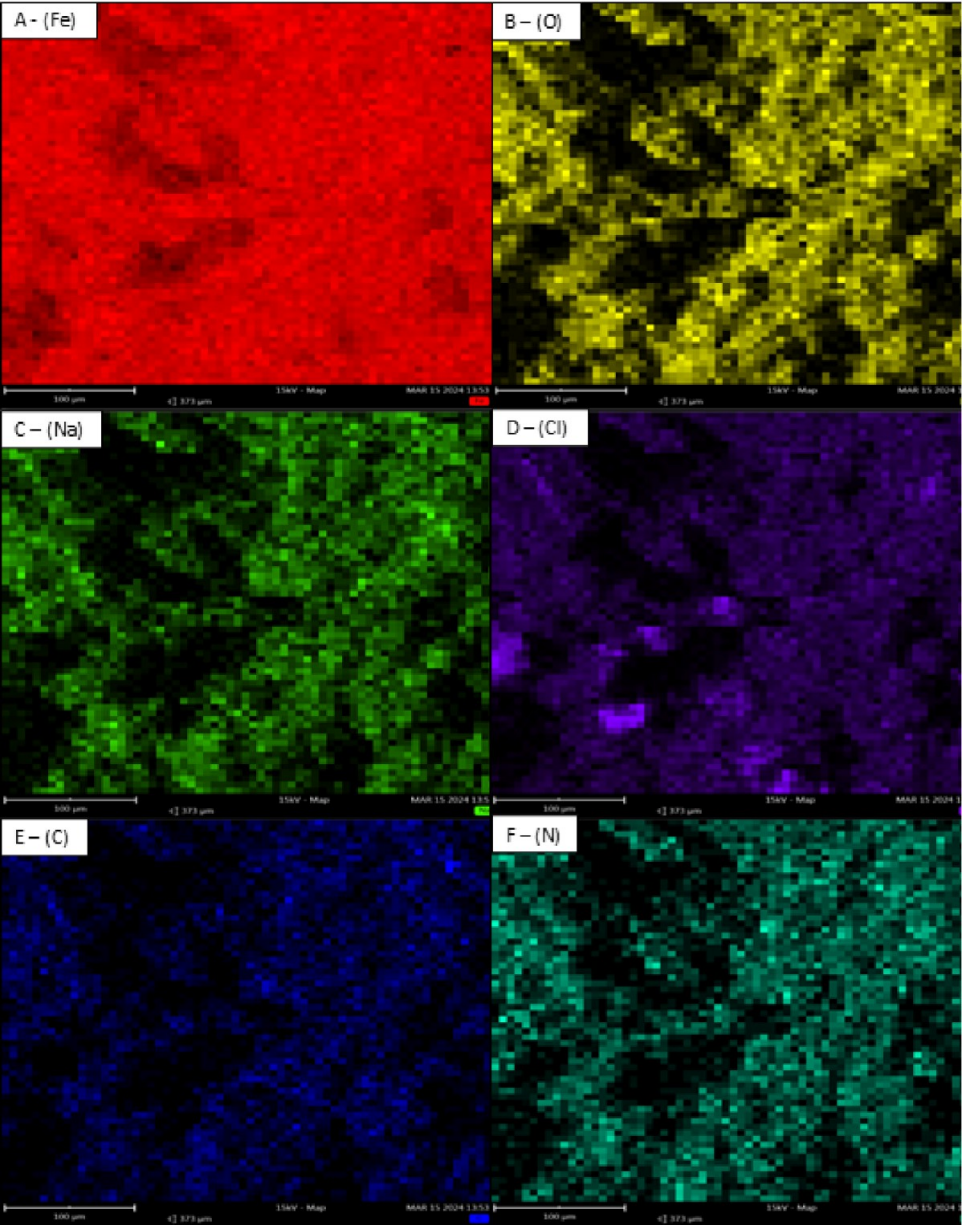
Energy-dispersive spectroscopy (EDS) elemental mapping of the A36 carbon steel surface after 24 h immersion in 3.5 wt % NaCl solution without corrosion inhibitors (blank condition): (A) Fe, (B) O, (C) Na, (D) Cl, (E) C, and (F) N.

**Table 8:** Elemental Composition of A36 Outside at the End of the 24-h Immersion Experiment for the Control Solution (NaCl wt 3.5%) with Corrosion Inhibitor (PIL A)

Element	Atomic Concentration	Weight Concentration
Fe	52.07	28.31
O	6.84	1.07
N	1.27	0.17
C	0.46	0.05
Cl	2.16	0.75
Na	0.21	0.05

**Figure 13:**
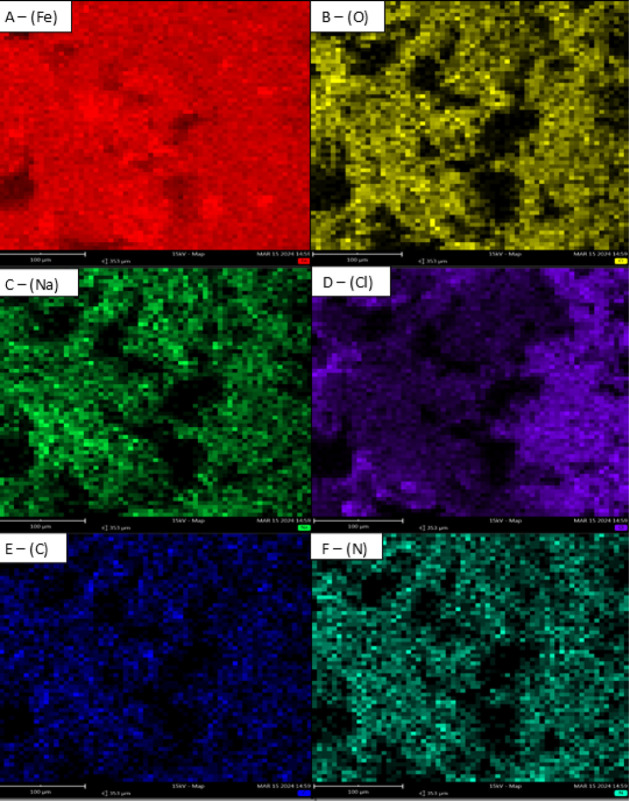
Energy-dispersive spectroscopy (EDS) elemental mapping of the A36 carbon steel surface after 24 h immersion in 3.5 wt % NaCl solution in the presence of the corrosion inhibitor PIL A (2-HDEAF): (A) Fe, (B) O, (C) Na, (D) Cl, (E) C, and (F) N.

The table above presents the elemental composition of A36 carbon steel exposed outdoors after a 24-h immersion in a control solution of 3.5% NaCl without any corrosion inhibitors (PILs). According to the data in Table 7, there is a noticeable increase in oxygen content, reaching approximately 7.5%. At the same time, the iron concentration on the surface decreases to 47.2%, while chlorine and nitrogen levels also drop to 0.82% and 0.71%, respectively.

These changes in elemental composition are linked to the increased formation of corrosion products such as lepidocrocite and magnetite, both of which contribute to the higher oxygen content detected on the metal surface. The rise in oxygen corresponds with a reduction in iron content, which falls below 50% as a consequence of oxide and oxyhydroxide formation.

The literature supports these findings by indicating that the presence of lepidocrocite and magnetite is closely associated with the corrosion product’s capacity to retain oxygen, thereby influencing the iron concentration observed on the steel surface. The formation of these phases results in the steel surface exhibiting differential retention of certain chemical elements, including chlorine.

This retention behavior is significant because chlorine accumulation can directly impact the material’s permeability and accelerate steel degradation over time. This phenomenon has critical implications for the long-term evaluation of metallic materials. The facilitated adsorption and retention of elements such as chlorine enhance their ability to penetrate the steel surface, promoting more aggressive corrosion processes.

Energy Dispersive Spectroscopy (EDS) analysis was instrumental in characterizing the steel exposed to the saline solution without corrosion inhibitors, providing detailed insight into the elemental distribution and corrosion behavior under these conditions.^118–120^ Figure 12 indicates element accumulation in distinct phases on the carbon steel surface after 24 h of exposure. This information is vital to assess how the formation of oxides/oxyhydroxides on the surface was detrimental.

Indeed, a predominant accumulation is observed across the entire material surface for the element iron, highlighted in red. The goal is to assess the iron oxides formed on carbon steel after applying the most effective corrosion inhibitors in a saline electrolyte. In contrast, certain areas show lower concentrations of iron and other elements.

These gaps are also visible in the EDS maps for oxygen, nitrogen, sodium, carbon, and chlorine. In addition, this result can be attributed to the fact that certain elements are more prevalent in specific iron oxides/oxyhydroxides when exposed to different conditions, pH, and salinity, with and without corrosion inhibitors.

Notably, the discussion on chloride permeation is particularly significant, as it was observed that there are points of chloride concentration in regions near this “vacancy”. This type of information still needs to be more extensive in literature, given that many articles mention chloride levels in higher concentrations. However, they have yet to investigate what type of corrosion products can play this function. Therefore, this article also examines the concentration factor of some elements on the steel surface after the corrosive process. These points often share typical iron oxide/oxyhydroxide phases, such as lepidocrocite and magnetite.

Image 13 presents six distinct images, each highlighting a different element in various colors. In Section A, iron (Fe), shown in red, predominates due to iron oxides and oxyhydroxides forming during the corrosive process. However, it is essential to note that certain areas exhibit lower concentrations of Fe, likely due to the formation of specific phases that readily absorb other elements, causing Fe to become secondary in the final composition.

The element oxygen, represented by the yellow color, has several regions with lesser concentrations, similar to the element chlorine, represented by the purple color. In addition, when evaluating the chlorine in the blank sample without a corrosion inhibitor, it can be detected that regions with higher chlorine concentrations on the sample are more distributed on the surface.

Evaluating the surface as a total area, it was found that these regions are associated with specific phases of iron oxides/oxyhydroxides. In the literature, the phases associated with this particular concentration are lepidocrocite and magnetite.^121^ The correlation between chloride accumulation in iron oxides/oxyhydroxides and the formation of different phases, such as lepidocrocite and maghemite, is influenced by the [Cl]/[Fe] molar ratio. As the ratio increases, the reaction time increases, which promotes the formation of lepidocrocite and reduces the area of the maghemite peak.

Chloride ions play a crucial role in the oxidation of iron, significantly influencing the stability and formation of corrosion precipitates. The phases present on the material’s surface exhibit a high chloride content, indicating which regions are more susceptible to degradation when exposed to saline environments over time.^118^

This susceptibility is partly due to the relatively weak adhesion of these phases to the metal surface, which results from weaker electrostatic interactions with the iron compared to those involving chloride ions.^118^ This observation suggests that the interaction between the sodium molecule and the iron surface is relatively weak compared to the strong electrostatic interaction between the chloride ion and the metal surface.

A high concentration of these elements does not characterize the presence of Nitrogen and Carbon on the surface. In contrast, there are more spaces when compared to elements such as Fe, O, Na, and Cl. For PIL A, the subsequent table shows the measured elemental composition of the material surface with the addition of corrosion inhibitors (PILs) after 24 h of immersion in saline solution (Table 8 and Figure 13).

The data analysis revealed an iron (Fe) concentration increase on carbon steel exposed to a 3.5 wt % saline solution with excess Cl^–^ and corrosion inhibitors, reaching 52%. At the same time, the chloride content was 2.16%. This effect can be attributed to the prolonged immersion time, which allows chloride ions to adhere more effectively to the surface, intensifying the corrosion process and increasing the release of Fe on the surface and into the solution.

Despite this relatively elevated value, no regions with a higher chloride concentration were observed, unlike in the blank sample. Instead, a more uniform chloride distribution was noted. This increase is significant as it is linked to a greater presence of lepidocrocite, especially in the regions to the right and at the edges of the image, where chloride absorption was more intense.

Furthermore, there was a reduction in oxygen and carbon concentrations, which decreased to 6.84% and 0.46%, respectively. The increase in nitrogen intensity to 1.27% is notable across the surface. It can be attributed to the presence of the inhibitor (PILs), which contains amines in its composition, suggesting an effective chemical interaction, as demonstrated by the author’s electrochemical and weight loss tests.

In contrast, the sodium concentration remained practically stable, likely due to the higher retention capacity of chloride in the analyzed structure.

In the first region, represented in red for Fe, a general predominance of iron is observed, as expected due to the formation of iron oxides/hydroxides on the surface. However, distinct areas of lower and higher iron concentrations were evident. This variation is likely due to the accumulation of oxides with differing iron content, resulting from the varied formation processes of corrosion products.

In the second region, represented in yellow for oxygen, the distribution closely mirrors that of nitrogen (N) in dark green and sodium (Na) in cyan. While these elements are generally distributed, notable gaps are likely caused by forming different oxides, each with varying capacities to absorb specific elements.

The variation in absorption capability results in the observed gaps in the distribution of these elements across the surface. The third region focuses on chlorine, highlighted in purple, which is generally well-distributed but with lower localized concentrations than the blank surface. This difference is likely due to the formation of specific iron oxides/hydroxides, such as goethite, which are denser and form a thicker surface film. This denser film can trap chlorine in localized areas, potentially creating concentrated permeation sites on the metal over time.

Nitrogen (N), marked in cyan, is evaluated in the fourth region. Nitrogen showed good overall distribution, with some gaps likely caused by oxide accumulation. The high nitrogen concentration across the surface may be attributed to the presence of amines in the corrosion inhibitor used to protect the material. This distribution pattern contrasts with the blank sample, where no inhibitors were added, highlighting the inhibitor’s influence on nitrogen distribution.

Finally, carbon showed a relatively lower distribution than other elements, indicating that carbon plays a less significant role in the overall elemental composition of the surface. The primary iron oxide phase associated with chloride concentration on the metal surface is lepidocrocite (γ-FeO­(OH)).

This phase threatens the long-term integrity of the material because chloride accelerates iron oxidation, resulting in corrosion products that undermine structural stability. Lepidocrocite is also susceptible to transforming into other phases, such as maghemite, which may be unstable and prone to further degradation. Additionally, the presence of chlorides creates a corrosive environment, exacerbating metal deterioration (Figure 13).

#### Mechanism of Inhibition

In recent decades, numerous chemical compounds have been used as corrosion inhibitors (CIs). In particular, ionic liquids (aprotic and protic), which are the focus of this study, with long alkyl chains between 5 and 18 carbon atoms, are known to have inhibitory properties due to their larger chain, which increases the insulation distance between the metal surface and the corrosive medium, thus protecting the material.^122^

Amusingly, the evaluation of ionic liquids with smaller carbon chains, usually up to six carbons, is in the minority in the literature, even though they show considerable positive results regarding inhibitory activity.^24^

Thus, careful evaluation of the experimental results (gravimetric, electrochemical, and surface) allows researchers to propose a possible mechanism of inhibition. First, they are believed to exert their inhibitory effect through the adsorption of both fractions (cations and anions) at the metal/solution electrochemical interface.^14,123^ Typically, adsorption is influenced by the structure and species,^24^ surface charge of the metal,^124^ and charge distribution over the entire inhibitor molecule.^78^ It is possible to understand the mechanism of inhibition of protic ionic liquids. Notably, oxidation and reduction reactions co-occur on the steel surface at both sites. They must be shown in the same picture for didactic purposes. First, the process at the cathodic sites is initiated by a reduction reaction (eq 4):

9 $$O_{2}+2H_{2}O+4e^{-}\to 4OH^{-}$$

The oxygen, presented in eq 6, resulting from an aerated medium, was successfully reduced in the electrochemical reduction, producing hydroxyl ions (OH^–^). This production was enhanced by the continuous flow of ions on the metallic surface, which resulted in a remarkable modification of the surface. Free electrons change from more active to less active sites (oxygen is converted to oxygen ions and combined with the four free electrons, which combine with water to form hydroxyl ions).^51^ The hydroxyl ions (OH^–^) formed by the reduction eq 4 are released into the solution and attracted to the cathodic region’s active surface. Consequently, this steel layer receives several hydroxyl ions (OH^–^), which form an attraction with the cation part of the protic ionic liquids and terminate the protective mechanism of the inhibitor in the cathodic region, as shown on the left side (Figure 14).

**Figure 14:**
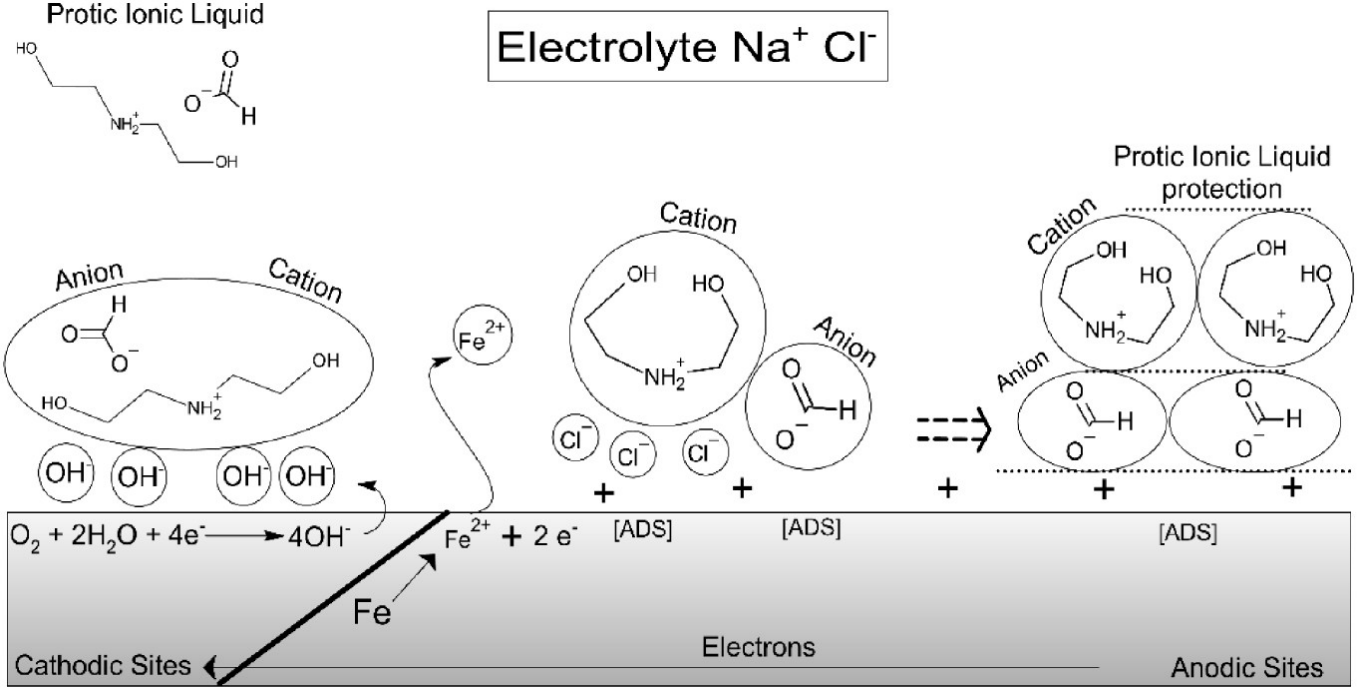
Mechanism of corrosion inhibition of carbon steel (A36) with LIPs in NaCl 3.5wt % (anodic and cathodic sites ).

At the anodic site, the dissolution of Fe(s) into Fe ions (Fe^2+^) in solution was observed because of the onset of the oxidation reaction. With the dissolution of iron into ions by the solution, the metal surface is positively attacked (+) owing to electrostatic transition, which attracts the chlorine anions in the solution to the surface and acts as a surface block.^47,125^

Given this attraction, there is an increase in the concentration of the most aggressive chemical fraction, Cl^–^ anions, on the metal surface, which is ascribed to the Coulomb forces. In addition to this discussion, this phenomenon promotes the hasty and effective adsorption of some molecules on the metallic surface, such as organic compounds, which are mainly positively charged owing to charge transfer and, in this case, attract the cationic fraction of protic ionic liquids, ensuring the inhibitory process after the formation of a protective barrier over the steel surface.^47,125^

Because of this attraction, the existing coupling of the fractions (cationic and anionic) of the protic ionic liquids on the steel surface is observed, which acts as a blocking method against external influences such as the corrosion process.^124^ In this protection, the cationic fraction of the ionic liquid is attracted by the adsorbed Cl^–^ ions on the surface, and the anionic fraction is attracted by the positively charged material after the dissolution of Fe^2+^ in the solution, creating a potential difference.^47,125^

Over time, the anionic fraction significantly influenced the metal surface by forming a protective layer akin to a simple coating. This outer layer facilitates better adaptation of the cationic fraction, which subsequently interacts with the adsorbed species to complete the formation of the final protective ionic liquid layer on the metal surface (Figure 14). All the LIPs evaluated in this study demonstrated promising corrosion inhibition performance, with efficiencies ranging from approximately 50% to 75%.

A key distinction among these compounds is their inherent sustainability and lack of bioaccumulation. The tested LIPs acted as mixed-type and anodic adsorption inhibitors. In particular, some exhibited a predominantly anodic inhibition mechanism, which promoted passive behavior at higher concentrations. For example, LIP A formed an adsorbed layer that effectively blocked the electrolyte’s attack on the steel substrate, enhancing the material’s resistance to corrosion

### Conclusion

In conclusion, this study demonstrates that the protic ionic liquids PIL A (2-HDEAF), PIL B (2-HDEAP), and PIL C (2-HDEAPe) act as effective corrosion inhibitors for ASTM A36 carbon steel in 3.5 wt % NaCl solution at room temperature (approximately 27–29 °C). Among the investigated compounds, PIL A exhibited the highest inhibition efficiency, likely due to its smaller acid fraction (formic acid) within the ionic liquid structure, followed by PIL B and PIL C (PIL A > PIL B > PIL C), with inhibition efficiencies exceeding 75% under optimal conditions. These results clearly highlight the critical role of molecular structure in governing corrosion inhibition performance.

Moreover, thermodynamic analysis based on Arrhenius and transition state models indicates that the adsorption of PILs on the steel surface is governed predominantly by a physisorption mechanism, characterized by endothermic dissolution and Δ*G*° _ads_ values close to −20 kJ mol^–1^. Adsorption isotherm analysis further revealed that the inhibition process follows the Frumkin model, emphasizing the role of lateral interactions between adsorbed inhibitor molecules. Such interactions significantly affect surface coverage and, consequently, the overall corrosion protection efficiency.

Physical adsorption is generally described in literature as being governed by relatively weak electrostatic interactions, which, although attractive, are not sufficiently strong to resist mechanical disturbances or surface wear. As a result, corrosion inhibition based predominantly on physisorption may lead to lower protection efficiencies when compared with systems dominated by stronger adsorption mechanisms, such as those often reported for aprotic ionic liquids. Nevertheless, despite these differences in inhibition strength, protic ionic liquids offer a distinct and important advantage due to their more sustainable and environmentally benign character, making them attractive alternatives for corrosion protection applications.

From an electrochemical standpoint, open-circuit potential (*E*_ocp_) measurements provided additional insight into the dynamic interactions between the PILs and the saline electrolyte under both stirred and unstirred conditions. The progressive shift of *E*_ocp_ toward more positive values upon PIL addition indicates the gradual formation of a protective interfacial layer, which subsequently evolves into a more stable corrosion-product based film on the carbon steel surface. At the optimal inhibitor concentration, a significant noble potential was observed compared to the uninhibited system, reflecting enhanced corrosion resistance.

Additionally, electrochemical polarization and impedance results consistently demonstrated the superior performance of PIL A.

Polarization curves revealed that PIL A acts predominantly as a mixed-type inhibitor, whereas PILs B and C exhibited a stronger anodic character. Impedance spectroscopy showed a systematic decrease in corrosion current density (*I*_corr_) and double-layer capacitance (*C*_dl_), alongside an increase in surface coverage (θ) and inhibition efficiency with increasing inhibitor concentration (250–1000 ppm). However, for PILs B and C, higher concentrations led to a deterioration in inhibition performance, likely associated with structural effects such as increased alkyl chain length, which may hinder efficient adsorption. Surface characterization by optical microscopy, SEM, and AFM corroborated the electrochemical findings, confirming that PIL A provides the most effective surface protection by minimizing corrosion-induced damage and surface roughness. These results underscore the importance of combining electrochemical techniques with complementary morphological analyses to elucidate inhibition mechanisms comprehensively. Completely, X-ray diffraction analysis identified corrosion products and oxide phases formed in the presence of PILs, providing direct evidence of the inhibition mechanism. The detection of phases such as goethite supports the proposed physisorption-driven protection process and reinforces the role of PIL A in stabilizing the steel surface under corrosive conditions. Overall, this work highlights the potential of structurally tailored protic ionic liquids as environmentally favorable and efficient corrosion inhibitors, offering valuable insights into the design of advanced interfacial protection systems.

## Supplementary Material



## Notes

Comprehensive physicochemical and structural characterization of the diethanolamine-based protic ionic liquids (PILs A, B, and C) investigated as corrosion inhibitors for carbon steel; measurements of dynamic and kinematic viscosity, density, electrical conductivity, pH in 3.5 wt % NaCl solution, refractive index, speed of sound, and moisture content, providing insight into ionic transport, molecular interactions, and solution behavior; detailed spectroscopic analyses, including ^1^H and ^13^C nuclear magnetic resonance (NMR) spectra confirming PIL formation via acid–base reactions and Fourier transform infrared (FTIR) spectra identifying functional groups and bonding environments; thermogravimetric analysis (TGA) data, including onset decomposition temperatures and maximum degradation rates, to evaluate the thermal stability of the PILs and their suitability for corrosion inhibition under varying temperature conditions; supplementary tables and figures to support comparative analysis among the synthesized PILs and to correlate physicochemical properties with electrochemical and surface characterization results discussed in the main manuscript (PDF)

## References

[1] Srivastava V., Haque J., Verma C., Singh P., Lgaz H., Salghi R., Quraishi M. A. (2017). Amino Acid Based Imidazolium Zwitterions
as Novel and Green Corrosion Inhibitors for Mild Steel: Experimental,
DFT and MD Studies. J. Mol. Liq..

[2] Bhardwaj N., Sharma P., Kumar V. (2021). Phytochemicals
as Steel Corrosion
Inhibitor: An Insight into Mechanism. Corros.
Rev..

[3] Reza N. A., Akhmal N. H., Fadil N. A., Taib M. F. M. (2021). A Review
on Plants
and Biomass Wastes as Organic Green Corrosion Inhibitors for Mild
Steel in Acidic Environment. Metals.

[4] Lopes J. C., Talon A. G., Rodrigues M. D. S., Moretti G. B., Machado F. D. C., Souza G. G. D., Ribeiro F. S. F., Sanchez L. E. D. A., Bianchi E. C. (2023). An Experimental
Evaluation between Pure and Diluted
MQL versus Flood Lubri-Cooling Focused on Cost and Environmental Impact. Int. J. Adv. Manuf. Technol..

[5] Escrivà-Cerdán C., Ooi S. W., Joshi G. R., Morana R., Bhadeshia H. K. D. H., Akid R. (2019). Effect of Tempering Heat Treatment on the CO2 Corrosion
Resistance of Quench-Hardened Cr-Mo Low-Alloy Steels for Oil and Gas
Applications. Corros. Sci..

[6] Khormali A., Ahmadi S., Aleksandrov A. N. (2025). Analysis
of Reservoir Rock Permeability
Changes Due to Solid Precipitation during Waterflooding Using Artificial
Neural Network. J. Pet. Explor. Prod. Technol..

[7] Ahmadi S., Khormali A., Kazemzadeh Y. (2025). A Critical
Review of the Phenomenon
of Inhibiting Asphaltene Precipitation in the Petroleum Industry. Processes.

[8] Khormali A., Ahmadi S., Kazemzadeh Y., Karami A. (2025). Evaluating the Efficacy
of Binary Benzimidazole Derivatives as Corrosion Inhibitors for Carbon
Steel Using Multi-Modal Analysis and Optimization Techniques. Results Eng..

[9] Shawabkeh R., Rihan R., Al-Baker N. (2013). Effect of an Alkyl Amine-Based Corrosion
Inhibitor for 1018 Carbon Steel Pipeline in Sea Water. Anti-Corros. Methods Mater..

[10] Ma Y., Li Y., Wang F. (2009). Corrosion
of Low Carbon Steel in Atmospheric Environments
of Different Chloride Content. Corros. Sci..

[11] Herrag L., Hammouti B., Elkadiri S., El Bali B., Lachkar M., Ouarsal R. (2008). Inhibition of Steel Corrosion in HCl Media by Phosphite
Compound. Pigment Resin Technol..

[12] Ortega
Vega M. R., Kunst S. R., da Silva J. A. T., Mattedi S., de Fraga Malfatti C. (2015). Influence of Anion Chain Length of Protic Ionic Liquids
on the Corrosion Resistance of API X70 Steel. Corros. Eng. Sci. Technol..

[13] Arellanes-Lozada P., Olivares-Xometl O., Likhanova N. V., Lijanova I. V., Vargas-García J. R., Hernández-Ramírez R. E. (2018). Adsorption and Performance of Ammonium-Based
Ionic Liquids as Corrosion Inhibitors of Steel. J. Mol. Liq..

[14] Schmitzhaus T. E., Ortega Vega M. R., Schroeder R., Muller I. L., Mattedi S., Malfatti C. D. F. (2020). An Amino-based
Protic Ionic Liquid as a Corrosion Inhibitor
of Mild Steel in Aqueous Chloride Solutions. Mater. Corros..

[15] Fernandes C. M., Alvarez L. X., dos Santos N. E., Maldonado Barrios A. C., Ponzio E. A. (2019). Green Synthesis of 1-Benzyl-4-Phenyl-1H-1,2,3-Triazole,
Its Application as Corrosion Inhibitor for Mild Steel in Acidic Medium
and New Approach of Classical Electrochemical Analyses. Corros. Sci..

[16] Younes N., Salem R., Al-Asmakh M., Altamash T., Pintus G., Khraisheh M., Nasrallah G. K. (2018). Toxicity Evaluation of Selected Ionic
Liquid Compounds on Embryonic Development of Zebrafish. Ecotoxicol. Environ. Saf..

[17] Peric B., Sierra J., Martí E., Cruañas R., Garau M. A., Arning J., Bottin-Weber U., Stolte S. (2013). (Eco)­Toxicity and Biodegradability of Selected Protic
and Aprotic Ionic Liquids. J. Hazard. Mater..

[18] Al-Tohamy R., Ali S. S., Li F., Okasha K. M., Mahmoud Y. A. G., Elsamahy T., Jiao H., Fu Y., Sun J. (2022). A Critical
Review on the Treatment of Dye-Containing Wastewater: Ecotoxicological
and Health Concerns of Textile Dyes and Possible Remediation Approaches
for Environmental Safety. Ecotoxicol. Environ.
Saf..

[19] Tzani A., Elmaloglou M., Kyriazis C., Aravopoulou D., Kleidas I., Papadopoulos A., Ioannou E., Kyritsis A., Voutsas E., Detsi A. (2016). Synthesis
and Structure-Properties
Relationship Studies of Biodegradable Hydroxylammonium-Based Protic
Ionic Liquids. J. Mol. Liq..

[20] Ge S., Chen L., Li W., Cai Q., Gong G., Wu J., Yu J., Nishimura K., Jiang N., Cai T. (2025). Tris-Based
Protic Ionic Liquids with Multiple Adsorption Effects Enabling Excellent
Anti-Wear and Anti-Corrosion Performances. J.
Mol. Liq..

[21] Sharma G., Gardas R. L., Coronas A., Venkatarathnam G. (2016). Effect of
Anion Chain Length on Physicochemical Properties of N,N-Dimethylethanolammonium
Based Protic Ionic Liquids. Fluid Phase Equilib..

[22] Markusson H., Belières J. P., Johansson P., Angell C. A., Jacobsson P. (2007). Prediction
of Macroscopic Properties of Protic Ionic Liquids by Ab Initio Calculations. J. Phys. Chem. A.

[23] Kumar P., Holmberg K., Soni I., Islam N., Kumar M., Shandilya P., Sillanpää M., Chauhan V. (2024). Advancements
in Ionic Liquid-Based Corrosion Inhibitors for Sustainable Protection
Strategies: From Experimental to Computational Insights. Adv. Colloid Interface Sci..

[24] Verma C., Ebenso E. E., Quraishi M. A. (2017). Ionic Liquids as
Green and Sustainable
Corrosion Inhibitors for Metals and Alloys: An Overview. J. Mol. Liq..

[25] Kobzar Y. L., Fatyeyeva K. (2021). Ionic Liquids as Green and Sustainable
Steel Corrosion
Inhibitors: Recent Developments. Chem. Eng.
J..

[26] Sharma J., Sharma S., Soni V. (2021). Classification and Impact of Synthetic
Textile Dyes on Aquatic Flora: A Review. Reg.
Stud. Mar. Sci..

[27] Brigouleix C., Anouti M., Jacquemin J., Caillon-Caravanier M., Galiano H., Lemordant D. (2010). Physicochemical
Characterization
of Morpholinium Cation Based Protic Ionic Liquids Used As Electrolytes. J. Phys. Chem. B.

[28] Ortega
Vega M. R., Ercolani J., Mattedi S., Aguzzoli C., Ferreira C. A., Rocha A. S., Malfatti C. F. (2018). Oleate-Based Protic
Ionic Liquids As Lubricants for Aluminum 1100. Ind. Eng. Chem. Res..

[29] Hulsbosch J., De Vos D. E., Binnemans K., Ameloot R. (2016). Biobased Ionic Liquids:
Solvents for a Green Processing Industry?. ACS
Sustain. Chem. Eng..

[30] Zhang J., Gong X. L., Yu H. H., Du M. (2011). The Inhibition Mechanism
of Imidazoline Phosphate Inhibitor for Q235 Steel in Hydrochloric
Acid Medium. Corros. Sci..

[31] Pascoal C. V. P., Da Silva L. R. R., Florez M. A. C., Cavalcante T. R. F., Avila J. A., Salomão F. C.
C. S., Barros E. B., Avelino F., Lomonaco D., Pinheiro R. S. (2025). Assessment
of Sustainable Ethanolamine-Based Protic Ionic Liquids with Varied
Carboxylic Acid Chains as Corrosion Inhibitors for Carbon Steel in
Saline Environments. Molecules.

[32] Pascoal C. V. P., da Silva G. B., da Silva D. S., Salomão F. C. C. S., Barros E. B., Vasques R. B., Pinheiro R. S., de Sant’ana H. B., Araújo W. S. (2024). Study of Protic Ionic Liquids as Sustained Corrosion
Inhibitors for Mild Steel in Saline Solution with Acidic PH and Temperature
Variations. Mater. Res..

[33] Chakravarthy M. P., Mohana K. N., Pradeep
Kumar C. B. (2014). Corrosion Inhibition Effect and Adsorption
Behaviour of Nicotinamide Derivatives on Mild Steel in Hydrochloric
Acid Solution. Int. J. Ind. Chem..

[34] Nesic S., Postlethwaite J., Olsen S. (1996). An Electrochemical Model for Prediction
of Corrosion of Mild Steel in Aqueous Carbon Dioxide Solutions. Corrosion.

[35] Reis C. L. B., Silva L. M. A. E., Rodrigues T. H. S., Félix A. K. N., Santiago-Aguiar R. S. D., Canuto K. M., Rocha M. V. P. (2017). Pretreatment of Cashew Apple Bagasse
Using Protic Ionic Liquids: Enhanced Enzymatic Hydrolysis. Bioresour. Technol..

[36] Pinheiro R. S., Mesquita F. M. R., Ponte M. S. R., Lopes L. C., Feitosa F. X., De Santiago-Aguiar R. S., De Sant’ana H. B. (2019). Liquid-Liquid
Equilibrium Data for Ternary Systems Containing Alkanes (n-Pentane,
n-Hexane, n-Heptane, and n-Octane) + Alcohol (Methanol and Ethanol)
+ Protic Ionic Liquid (2-HEAF). J. Chem. Eng.
Data.

[37] Somers A. E., Hinton B. R. W., de Bruin-Dickason C., Deacon G. B., Junk P. C., Forsyth M. (2018). New, environmentally
friendly, Rare Earth Carboxylate
Corrosion Inhibitors for Mild Steel. Corros.
Sci..

[38] Burkle D., De Motte R., Taleb W., Kleppe A., Comyn T., Vargas S. M., Neville A., Barker R. (2017). In Situ SR-XRD Study
of FeCO3 Precipitation Kinetics onto Carbon Steel in CO2-Containing
Environments: The Influence of Brine PH. Electrochim.
Acta.

[39] Zhang H. H., Gao K., Yan L., Pang X. (2017). Inhibition of the Corrosion of X70
and Q235 Steel in CO2-Saturated Brine by Imidazoline-Based Inhibitor. J. Electroanal. Chem..

[40] Iglesias M., Gonzalez-Olmos R., Cota I., Medina F. (2010). Brønsted Ionic
Liquids: Study of Physico-Chemical Properties and Catalytic Activity
in Aldol Condensations. Chem. Eng. J..

[41] Johnson K. E., Driver G. W. (2010). Protons: Affinity and Reduction in Ionic Liquids. ECS Trans..

[42] Alvarez V. H., Mattedi S., Martin-Pastor M., Aznar M., Iglesias M. (2011). Thermophysical
Properties of Binary Mixtures of {ionic Liquid 2-Hydroxy Ethylammonium
Acetate + (Water, Methanol, or Ethanol)}. J.
Chem. Thermodyn..

[43] Pineda F., Walczak M., Vilchez F., Guerra C., Escobar R., Sancy M. (2022). Evolution of Corrosion
Products on ASTM A36 and AISI 304L Steels
Formed in Exposure to Molten NaNO3–KNO3 Eutectic Salt: Electrochemical
Study. Corros. Sci..

[44] Almeida T. D. C., Bandeira M. C. E., Moreira R. M., Mattos O. R. (2017). New Insights
on the Role of CO2 in the Mechanism of Carbon Steel Corrosion. Corros. Sci..

[45] Moura M. J. S., Vasques R. B., Levy M. M., Magalhães S. J. M., Pascoal C. V. P., Almeida-Neto F. W. Q., Lima-Neto P., Medeiros S. L. S., Salomão F. C. C. S., Barros E. B., Araújo W. S. (2024). Study of the Efficiency of the Amino
Acid L-Histidine as a Corrosion Inhibitor of 1018 Carbon Steel in
Saline Solution Without and with CO2 Saturation. Mater. Res..

[46] de
M. Fideles F. F., Florez M. A. C., Rodrigues M. V. G., Cardoso J. L., Aranas C., Rodrigues S. F., da S. Lima M. N., Pascoal C. V. P., de Moura T. A., Reis G. S. (2023). Influence of the Morphology of Eutectoid Steels on Corrosion Resistance
in NaCl Aqueous Medium with and without CO2. Metals.

[47] Munis A., Zhao T., Zheng M., Rehman A. U., Wang F. (2020). A Newly Synthesized
Green Corrosion Inhibitor Imidazoline Derivative for Carbon Steel
in 7.5% NH4Cl Solution. Sustainable Chem. Pharm..

[48] Nwanya A. C., Ndipingwi M. M., Mayedwaa N., Razanamahandry L. C., Ikpo C. O., Waryo T., Ntwampe S. K. O., Malenga E., Fosso-Kankeu E., Ezema F. I., Iwuoha E. I., Maaza M. (2019). Maize (Zea
Mays L.) Fresh Husk Mediated Biosynthesis of Copper Oxides: Potentials
for Pseudo Capacitive Energy Storage. Electrochim.
Acta.

[49] Costa S. N., Almeida-Neto F. W. Q., Campos O. S., Fonseca T. S., de Mattos M. C., Freire V. N., Homem-de-Mello P., Marinho E. S., Monteiro N. K. V., Correia A. N., de Lima-Neto P. (2021). Carbon Steel Corrosion Inhibition
in Acid Medium by Imidazole-Based Molecules: Experimental and Molecular
Modelling Approaches. J. Mol. Liq..

[50] Kumar P., Kalia V., Kumar H., Dahiya H. (2017). Corrosion Inhibition
for Mild Steel in Acidic Medium by Using Hexadecylamine as Corrosion
Inhibitor. Chem. Sci. Trans..

[51] Verma C., Ebenso E. E., Quraishi M. A. (2017). Corrosion Inhibitors
for Ferrous
and Non-Ferrous Metals and Alloys in Ionic Sodium Chloride Solutions:
A Review. J. Mol. Liq..

[52] Martín L. A., Popovich C. A., Martinez A. M., Damiani M. C., Leonardi P. I. (2016). Oil Assessment
of Halamphora Coffeaeformis Diatom Growing in a Hybrid Two-Stage System
for Biodiesel Production. Renew. Energy.

[53] Wang X., Huang A., Lin D., Talha M., Lin Y., Liu H. (2020). Imidazolium-Based Ionic
Liquid as Efficient Corrosion Inhibitor for
Aa 6061 Alloy in Hcl Solution. Materials.

[54] Yousefi A., Javadian S., Dalir N., Kakemam J., Akbari J. (2015). Imidazolium-Based
Ionic Liquids as Modulators of Corrosion Inhibition of SDS on Mild
Steel in Hydrochloric Acid Solutions: Experimental and Theoretical
Studies. RSC Adv..

[55] El-Saeed H. M., Fouda A. S., Deyab M. A., Shalabi K., Nessim M. I., El-Katori E. E. (2022). Synthesis
and Characterization of Novel Ionic Liquids
Based on Imidazolium for Acid Corrosion Inhibition of Aluminum: Experimental,
Spectral, and Computational Study. J. Mol. Liq..

[56] El-Nagar R. A., Khalil N. A., Atef Y., Nessim M. I., Ghanem A. (2024). Evaluation
of Ionic Liquids Based Imidazolium Salts as an Environmentally Friendly
Corrosion Inhibitors for Carbon Steel in HCl Solutions. Sci. Rep..

[57] Verma C., Alrefaee S. H., Quraishi M. A. A., Ebenso E. E., Hussain C. M. (2021). Recent
Developments in Sustainable Corrosion Inhibitors Using Ionic Liquids:
A Review. J. Mol. Liq..

[58] Nascimento R. C., Furtado L. B., Guimarães M. J.
O. C., Seidl P. R., Rocha J. C., Ponciano J. A. C., Cruz M. T. M. (2018). Synergistic Effect
of Propargyl Alcohol, Octadecylamine, and 1,3-Dibutyl Thiourea for
API P110 Alloys in Acetic and Formic Acidic Solutions Used in Oil
Well Acidizing. J. Mol. Liq..

[59] Akanji O. L., Loto C. A., Popoola A. P. I. (2017). A Study
of the Electrochemical Behaviour
of Mild Steel in Potassium Gluconate-0.5 M Hydrochloric Acid Solution:
Grey Relational Model. Int. J. Adv. Manuf. Technol..

[60] Corrales-Luna M., Le Manh T., Romero-Romo M., Palomar-Pardavé M., Arce-Estrada E. M. (2019). 1-Ethyl
3-Methylimidazolium Thiocyanate Ionic Liquid
as Corrosion Inhibitor of API 5L X52 Steel in H2SO4 and HCl Media. Corros. Sci..

[61] Castaño J. G., Botero C. A., Restrepo A. H., Agudelo E. A., Correa E., Echeverría F. (2010). Atmospheric Corrosion of Carbon Steel
in Colombia. Corros. Sci..

[62] Islam M. M., Pojtanabuntoeng T., Gubner R. (2018). Corrosion of Carbon Steel under Condensing
Water and Monoethylene Glycol. Corros. Sci..

[63] Wei L., Pang X., Gao K. (2016). Corrosion of Low Alloy Steel and
Stainless Steel in Supercritical CO 2/H 2 O/H 2 S Systems. Corros. Sci..

[64] Peric B., Martí E., Sierra J., Cruañas R., Iglesias M., Garau M. A. (2011). Terrestrial Ecotoxicity of Short
Aliphatic Protic Ionic Liquids. Environ. Toxicol.
Chem..

[65] Vega M. R. O., Parise K., Ramos L. B., Boff U., Mattedi S., Schaeffer L., Malfatti C. F. (2017). Protic Ionic Liquids Used as Metal-Forming
Green Lubricants for Aluminum: Effect of Anion Chain Length. Mater. Res..

[66] Silva C. C., Miranda H. C., de Sant’ana H.
B., Farias J. P. (2013). Austenitic
and Ferritic Stainless Steel Dissimilar Weld Metal Evaluation for
the Applications As-Coating in the Petroleum Processing Equipment. Mater. Des..

[67] Corrales
Luna M., Le Manh T., Cabrera Sierra R., Medina Flores J. V., Lartundo Rojas L., Arce Estrada E. M. (2019). Study of Corrosion Behavior of API
5L X52 Steel in Sulfuric Acid in the Presence of Ionic Liquid 1-Ethyl
3-Methylimidazolium Thiocyanate as Corrosion Inhibitor. J. Mol. Liq..

[68] Al-Baker N., Shawabkeh R., Rihan R. (2011). Kinetic Study of Effect of Amine
Based Corrosion Inhibitor in Reducing Corrosion Rate of 1018 Carbon
Steel in Seawater Solution. Corros. Eng. Sci.
Technol..

[69] Al-Sarawy A. A., Fouda A. S., El-Dein W. A. S. (2008). Some
Thiazole Derivatives as Corrosion
Inhibitors for Carbon Steel in Acidic Medium. Desalination.

[70] Martins L. F., Cubides-Román D. C., da Silveira V. C., Aquije G. M. F. V., Romão W., dos Santos R. B., Neto Á. C., Lacerda V. (2020). Synthesis of
New Phenolic-Schiff Base and Its Application as Antioxidant in Soybean
Biodiesel and Corrosion Inhibitor in AISI 1020 Carbon Steel. J. Braz. Chem. Soc..

[71] Peimani A., Nasr-Esfahani M. (2018). Application of Anise Extract for
Corrosion Inhibition
of Carbon Steel in CO2 Saturated 3.0% NaCl Solution. Prot. Met. Phys. Chem. Surfaces.

[72] Morcillo M., De La Fuente D., Díaz I., Cano H. (2011). Atmospheric Corrosion
of Mild Steel. Rev. Metal..

[73] Umoren S. A., Solomon M. M., Obot I. B., Sulieman R. K. (2019). A Critical Review
on the Recent Studies on Plant Biomaterials as Corrosion Inhibitors
for Industrial Metals. J. Ind. Eng. Chem..

[74] Bajares R. A., Di Mella L. (2015). Study of the Corrosion
Rate in the Couple of Steels
ASTM A-36 and AISI/SAE 304 in a Water-Coke of Petroleum System. Procedia Mater. Sci..

[75] Bhattacharjee A., Coutinho J. A. P., Freire M. G., Carvalho P. J. (2015). Thermophysical
Properties
of Two Ammonium-Based Protic Ionic Liquids. J. Solution Chem..

[76] Abbasov V. M., Abd El-Lateef H. M., Aliyeva L. I., Qasimov E. E., Ismayilov I. T., Khalaf M. M. (2013). A Study of the Corrosion Inhibition of Mild Steel C1018
in CO2-Saturated Brine Using Some Novel Surfactants Based on Corn
Oil. Egypt. J. Pet..

[77] Javadian S., Darbasizadeh B., Yousefi A., Ektefa F., Dalir N., Kakemam J. (2017). Dye-Surfactant
Aggregates as Corrosion Inhibitor for
Mild Steel in NaCl Medium: Experimental and Theoretical Studies. J. Taiwan Inst. Chem. Eng..

[78] Solmaz R. (2010). Investigation
of the Inhibition Effect of5-((E)-4-Phenylbuta-1,3-Dienylideneamino)-1,3,4-Thiadiazole-2-Thiol
Schiff Base on Mild Steel Corrosion in Hydrochloric Acid. Corros. Sci..

[79] Amin M. A. W. L. (2006). Polarization
Electrochemical Impedance Spectroscopy, SEM and EDX Studies of the
Corrosion Inhibition of Copper in Aerated NaCl Solutions. J. Appl. Electrochem..

[80] Ramya K., Anupama K. K., Shainy K. M., Joseph A. (2017). Corrosion Protection
of Mild Steel in Hydrochloric Acid Solution through the Synergistic
of Alkylbenzimidazoles and Semicarbazide Pair – Electroanalytical
and Computational Studies. Egypt. J. Pet..

[81] Popoola L. T. (2019). Organic
Green Corrosion Inhibitors (OGCIs): A Critical Review. Corros. Rev..

[82] Zeng X., Zheng X., Guo L., Xu Q., Huang H., Tan B. (2021). Three Imidazole Ionic Liquids as
Green and Eco-Friendly Corrosion
Inhibitors for Mild Steel in Sulfuric Acid Medium. J. Mol. Liq..

[83] Subasree N., Selvi J. A. (2020). Imidazolium Based Ionic Liquid Derivatives; Synthesis
and Evaluation of Inhibitory Effect on Mild Steel Corrosion in Hydrochloric
Acid Solution. Heliyon.

[84] Zeino A., Abdulazeez I., Khaled M., Jawich M. W., Obot I. B. (2018). Mechanistic
Study of Polyaspartic Acid (PASP) as Eco-Friendly Corrosion Inhibitor
on Mild Steel in 3% NaCl Aerated Solution. J.
Mol. Liq..

[85] Ardakani E. K., Kowsari E., Ehsani A., Ramakrishna S. (2021). Performance
of All Ionic Liquids as the Eco-Friendly and Sustainable Compounds
in Inhibiting Corrosion in Various Media: A Comprehensive Review. Microchem. J..

[86] El-Tabei A. S., El-Tabey A. E., El Basiony N. M. (2022). Newly Imine-Azo Dicationic Amphiphilic
for Corrosion and Sulfate-Reducing Bacteria Inhibition in Petroleum
Processes: Laboratory and Theoretical Studies. Appl. Surf. Sci..

[87] Verma C., Olasunkanmi L. O., Obot I. B., Ebenso E. E., Quraishi M. A. (2016). 5-Arylpyrimido-[4,5-b]­Quinoline-Diones
as New and Sustainable Corrosion Inhibitors for Mild Steel in 1 M
HCl: A Combined Experimental and Theoretical Approach. RSC Adv..

[88] Aslam R., Mobin M., Huda, Obot I. B., Alamri A. H. (2020). Ionic Liquids
Derived
from α-Amino Acid Ester Salts as Potent Green Corrosion Inhibitors
for Mild Steel in 1M HCl. J. Mol. Liq..

[89] Verma C., Obot I. B., Bahadur I., Sherif E.-S. M., Ebenso E. E. (2018). Choline
Based Ionic Liquids as Sustainable Corrosion Inhibitors on Mild Steel
Surface in Acidic Medium: Gravimetric, Electrochemical, Surface Morphology,
DFT and Monte Carlo Simulation Studies. Appl.
Surf. Sci..

[90] Abdel-Gaber A. M., Abd-El-Nabey B. A., Khamis E., Abd-El-Khalek D. E. (2011). A Natural
Extract as Scale and Corrosion Inhibitor for Steel Surface in Brine
Solution. Desalination.

[91] Guo Y., Xu B., Liu Y., Yang W., Yin X., Chen Y., Le J., Chen Z. (2017). Corrosion Inhibition Properties of Two Imidazolium
Ionic Liquids with Hydrophilic Tetrafluoroborate and Hydrophobic Hexafluorophosphate
Anions in Acid Medium. J. Ind. Eng. Chem..

[92] Pourghasemi
Hanza A., Naderi R., Kowsari E., Sayebani M. (2016). Corrosion
Behavior of Mild Steel in H2SO4 Solution with 1,4-Di [1′-Methylene-3′-Methyl
Imidazolium Bromide]-Benzene as an Ionic Liquid. Corros. Sci..

[93] Ismail K. M. (2007). Evaluation
of Cysteine as Environmentally Friendly Corrosion Inhibitor for Copper
in Neutral and Acidic Chloride Solutions. Electrochim.
Acta.

[94] Fekry A. M., Mohamed R. R. (2010). Acetyl Thiourea
Chitosan as an Eco-Friendly Inhibitor
for Mild Steel in Sulphuric Acid Medium. Electrochim.
Acta.

[95] El
Hajjaji F., Salim R., Taleb M., Benhiba F., Rezki N., Chauhan D. S., Quraishi M. A. (2021). Pyridinium-Based
Ionic Liquids as Novel Eco-Friendly Corrosion Inhibitors for Mild
Steel in Molar Hydrochloric Acid: Experimental & Computational
Approach. Surf. Interfaces.

[96] Goyal M., Vashisht H., Kumar S., Bahadur I. (2018). Anti-Corrosion Performance
of Eco-Friendly Inhibitor (2-Aminobenzyl) Triphenylphosphonium Bromide
Ionic Liquid on Mild Steel in 0.5 M Sulfuric Acid. J. Mol. Liq..

[97] Desimone M. P., Gordillo G., Simison S. N. (2011). The Effect of Temperature
and Concentration
on the Corrosion Inhibition Mechanism of an Amphiphilic Amido-Amine
in CO2 Saturated Solution. Corros. Sci..

[98] Leibler L., Orland H., Wheeler J. C. (1983). Theory of Critical Micelle Concentration
for Solutions of Block Copolymers. J. Chem.
Phys..

[99] Fuchs-Godec R. (2006). The Adsorption,
CMC Determination and Corrosion Inhibition of Some N-Alkyl Quaternary
Ammonium Salts on Carbon Steel Surface in 2 M H2SO4. Colloids Surf., A.

[100] Kowsari E., Payami M., Amini R., Ramezanzadeh B., Javanbakht M. (2014). Task-Specific Ionic Liquid as a New
Green Inhibitor
of Mild Steel Corrosion. Appl. Surf. Sci..

[101] Santana Rodríguez J. J., Santana
Hernández F. J., González González J. E. (2002). XRD and
SEM Studies of the Layer
of Corrosion Products for Carbon Steel in Various Different Environments
in the Province of Las Palmas (The Canary Islands, Spain). Corros. Sci..

[102] Varvara S., Bostan R., Bobis O., Găină L., Popa F., Mena V., Souto R. M. (2017). Propolis
as a Green
Corrosion Inhibitor for Bronze in Weakly Acidic Solution. Appl. Surf. Sci..

[103] Zunita M., Kevin Y. J. (2022). Ionic Liquids as Corrosion Inhibitor:
From Research and Development to Commercialization. Results Eng..

[104] Fan Y., Liu W., Li S., Chowwanonthapunya T., Wongpat B., Zhao Y., Dong B., Zhang T., Li X. (2020). Evolution of Rust Layers
on Carbon Steel and Weathering Steel in
High Humidity and Heat Marine Atmospheric Corrosion. J. Mater. Sci. Technol..

[105] Antunes R. A., Costa I., de Faria D. L. A. (2003). Characterization
of Corrosion Products Formed on Steels in the First Months of Atmospheric
Exposure. Mater. Res..

[106] Xiao H., Ye W., Song X., Ma Y., Li Y. (2017). Evolution of Akaganeite in Rust Layers Formed on Steel
Submitted
to Wet/Dry Cyclic Tests. Materials.

[107] de la Fuente D., Alcántara J., Chico B., Díaz I., Jiménez J. A., Morcillo M. (2016). Characterisation of Rust Surfaces
Formed on Mild Steel Exposed to Marine Atmospheres Using XRD and SEM/Micro-Raman
Techniques. Corros. Sci..

[108] Balboni E., Smith K. F., Moreau L. M., Li T. T., Maloubier M., Booth C. H., Kersting A. B., Zavarin M. (2020). Transformation
of Ferrihydrite to Goethite and the Fate of Plutonium. ACS Earth Sp. Chem..

[109] Das S., Hendry M. J., Essilfie-Dughan J. (2011). Transformation
of Two-Line Ferrihydrite
to Goethite and Hematite as a Function of PH and Temperature. Environ. Sci. Technol..

[110] Deng Y. (1997). Formation of Iron­(III) Hydroxides
from Homogeneous Solutions. Water Res..

[111] Pedrosa J., Costa B. F. O., Portugal A., Durães L. (2015). Controlled
Phase Formation of Nanocrystalline Iron Oxides/Hydroxides in Solution
– An Insight on the Phase Transformation Mechanisms. Mater. Chem. Phys..

[112] Nolasco-Sobrinho P. J., Espinosa D. C. R., Tenório J. A. S. (2003). Characterisation
of Dusts and Sludges Generated during Stainless Steel Production in
Brazilian Industries. Ironmak. Steelmak..

[113] Cornell, R. M. ; Schwertmann, U. The Iron Oxides; Wiley, 2003. DOI: 10.1002/3527602097.

[114] Fonna S., Ibrahim I. B. M., Gunawarman, Huzni S., Ikhsan M., Thalib S. (2021). Investigation
of Corrosion Products Formed on the Surface
of Carbon Steel Exposed in Banda Aceh’s Atmosphere. Heliyon.

[115] Raman A., Razvan A., Kuban B., Clement K. A., Graves W. E. (1986). Characteristics of the Rust From Weathering Steels
in Louisiana Bridge Spans. Corrosion.

[116] Raman A., Nasrazadani S., Sharma L. (1989). Morphology of Rust
Phases Formed on Weathering Steels in Various Laboratory Corrosion
Tests. Metallography.

[117] Alcántara J., Chico B., Simancas J., Díaz I., de la Fuente D., Morcillo M. (2016). An Attempt to Classify
the Morphologies
Presented by Different Rust Phases Formed during the Exposure of Carbon
Steel to Marine Atmospheres. Mater. Charact..

[118] Taylor R. M. (1984). Influence of Chloride on the Formation
of Iron Oxides
From Fe­(II) Chloride. I. Effect of [Cl]/[Fe] on the Formation of Magnetite. Clays Clay Miner..

[119] Borjali S., Allahkaram S. R., Khosravi H. (2012). Effects of Working
Temperature and Carbon Diffusion on the Microstructure of High Pressure
Heat-Resistant Stainless Steel Tubes Used in Pyrolysis Furnaces during
Service Condition. Mater. Des..

[120] Zhang X., Leygraf C., Odnevall
Wallinder I. (2013). Atmospheric
Corrosion of Galfan Coatings on Steel in Chloride-Rich Environments. Corros. Sci..

[121] Zhu Y., Free M. L., Woollam R., Durnie W. (2017). A Review of
Surfactants
as Corrosion Inhibitors and Associated Modeling. Prog. Mater. Sci..

[122] Olivares-Xometl O., Álvarez-Álvarez E., Likhanova N. V., Lijanova I. V., Hernández-Ramírez R. E., Arellanes-Lozada P., Varela-Caselis J. L. (2018). Synthesis and Corrosion Inhibition
Mechanism of Ammonium-Based Ionic Liquids on API 5L X60 Steel in Sulfuric
Acid Solution. J. Adhes. Sci. Technol..

[123] Schmitzhaus T. E., Ortega Vega M. R., Schroeder R., Muller I. L., Mattedi S., Malfatti C. D. F. (2020). N -methyl-2-hydroxyethylammonium
Oleate Ionic Liquid Performance as Corrosion Inhibitor for Mild Steel
in Hydrochloric Acid Medium. Mater. Corros..

[124] Abbas M. A., Zakaria K., El-Shamy A. M., Abedin S. Z. E. (2021). Utilization
of 1-Butylpyrrolidinium Chloride Ionic Liquid as an Eco-Friendly Corrosion
Inhibitor and Biocide for Oilfield Equipment: Combined Weight Loss,
Electrochemical and SEM Studies. Z. Phys. Chem..

[125] Lin B., Tang J., Wang Y., Wang H., Zuo Y. (2020). Study on Synergistic
Corrosion Inhibition Effect between Calcium Lignosulfonate (CLS) and
Inorganic Inhibitors on Q235 Carbon Steel in Alkaline Environment
with Cl–. Molecules.

