# Supplementary material for: Understanding the Role of Diethanolamine-Based Protic Ionic Liquids in Corrosion Inhibition: Electrochemical and Surface Characterization of Carbon Steel in Saline Environments
Source: Langmuir. 2026 Jan 21;42(4):3417–43. doi: 10.1021/acs.langmuir.5c05642 (PMC12874530; doi:10.1021/acs.langmuir.5c05642)
Supplement: Supplementary file 1 [file la5c05642_si_001.pdf]

Supporting Information (SI)

Understanding the role of diethanolamine-based protic ionic liquids in corrosion inhibition: electrochemical and surface characterization of carbon steel in saline environments.

Caio Victor Pereira Pascoal [a, *], Mauro Andres Cerra Florez [a], Francisco Carlos Carneiro Soares Salomão [b], Eduardo Bedê Barros [c], Regiane Silva Pinheiro [d], Mohammad Rezayat [f, g], Gemma Fargas [f, g], Hosiberto Batista de Sant'Ana, H.B [e], Walney Silva Araújo [a, **].

[a] Department of Metallurgical and Materials Engineering, Federal University of Ceará (UFC), Fortaleza/CE, 60.440-900, Brazil.
[b] Ceara State University (UECE), Science and Technology Center. Fortaleza/CE, 60.714-903, Brazil.
[c] Department of Physics, Federal University of Ceará (UFC), Fortaleza/CE, 60.455-760 Brazil
[d] Department of Food Engineering Federal University of Maranhão (UFMA), Imperatriz, Ma, 65.915-060, Brazil
[e] Department of Chemical Engineering, Federal University of Ceará Fortaleza (UFC), CE, 60.440-554, Brazil.
[f] Department of Materials Science and Engineering, (EEBE), Universitat Politècnica de Catalunya, (CIEFMA), UPC, 08019, Barcelona, Spain
[g] Barcelona Research Center in Multiscale Science and Engineering, Universitat Politècnica de Catalunya, UPC, 08019, Barcelona, Spain.

*/**Corresponding Authors: E-mail address: caiovictorppascoal@gmail.com (Caio Victor) / wsa@ufc.br (Walney Silva Araújo).

Supporting Information Table of Contents

- **Physicochemical properties of diethanolamine-based protic ionic liquids (PILs A–C)**
  – Table S1 - Density, dynamic and kinematic viscosity, electrical conductivity, pH, refractive index, speed of sound, and moisture content.
- **Nuclear magnetic resonance spectroscopy**
  – Figure S1. 1H NMR spectra of PILs A–C
  – Figure S2. 13C NMR spectra of PILs A–C
- **Fourier transform infrared spectroscopy**
  – Figure S3. FTIR spectra of PILs A–C
- **Thermogravimetric analysis**
  – Figure S4 - TGA curves of PILs A–C
  – Table S2 - Onset and maximum decomposition temperatures of PILs

Supporting Information

We confirm that all images, artwork, and photographs included in the manuscript and in the Supporting Information, including the Table of Contents (TOC) graphics, were created by the authors of this manuscript and do not infringe upon any third-party copyrights.

We confirm that the Table of Contents (TOC) graphic is an original work created exclusively by the authors of this manuscript and does not contain any third-party material.

Protic Ionic Liquids (PILs) characterization

To understand the electrochemical behavior of protic ionic liquids on metallic surfaces in solution, a complete and comprehensive physicochemical study of their characteristics was conducted. The dynamic and kinematic viscosity, density, conductivity on the NaCl solution, pH, sound velocity, refractive index, and moisture content were evaluated (**Table S1**) [1].

Table S1
Protic ionic liquid characterization (abbreviations, structures, supplier molecules, density, viscosity, conductivity, and pH of the six PIL-synthesized inhibitors).

PILs	V. Dynamic (mPa.S) *	V. Kinematic (mm^2/S) *	Molar Mass	Dens.	*Cond	*pH	Speed of sound (m/s)	Refractive Index (nD)	Moisture (%)
PIL-A	47.73	40.70	151.0	1.1702	13.40	8.4	1867	1.45	7.26
PIL-B	270.0	237.5	178.1	1.1368	16.96	6.7	1799	1.46	4.73
PIL-C	169.3	156.7	206.1	1.0801	16.95	6.5	1678	1.45	5.30

*NaCl 3.5% pH and conductivity.

There are two types of viscosity: dynamic and kinematic. Dynamic viscosity (μ) can be summarized as the force required to move a unit area of a given compound over a unit distance. Kinematic viscosity is calculated as the value of dynamic viscosity divided by the density of the fluid being studied. Both characterization methods provide information related to the kinetic behavior of the evaluated molecule

For dynamic viscosity values, it is well known that as the carbon chain length increases in the acids used in this study, the viscosity values increase proportionally, ranging from 23.94 to 102.4 mPa·s. To confirm this, kinematic viscosity was also measured, and the same trend was observed, with values ranging from 20.76 to 97.46 mm^2/s, confirming the correlation between dynamic and kinematic viscosity. Correspondingly, as viscosity values increased, the density values decreased with the increase in the carbon chain length of the PILs tested, from 1.15 to 1.05 g/cm^3 [2–6]. In corrosion inhibitor studies, the evaluation of conductivity and pH in a NaCl (3.5%) solution is directly related, as the addition of a certain amount of these compounds to the solution is not intended to reduce pH or conductivity [1,7].

Certain thermodynamic characterizations of chemical systems and interactions between corrosion inhibitors and electrolytes can explain specific macroscopic properties. In particular, evaluating the density and speed of sound by varying the chemical components (PILs) allows for a deeper understanding of the chemical interactions among all species in solution for corrosion prevention purposes [8].

In accordance with Keshapolla et al. [8], sound waves interacting with the evaluated materials (PILs) provide accurate information about molecular phenomena, inhibitors, and the steel surface. The speed of sound of the studied PILs was measured under these conditions (**Table S1**). The results indicate that with increasing chain length, particularly in the acids, whether in cations or anions, the speed of sound increased. This observation is useful for evaluating the suitability of certain chemicals for specific applications, such as corrosion prevention.

However, the results show a relatively different situation regarding the study of PILs, as there was a reduction in the speed of sound with an increase in the carbon chain length. This outcome can be attributed to the nature of the ionic liquids investigated, as PILs belong to a different structural class and exhibit properties distinct from those of AILs. Furthermore, the speed of sound values obtained for the PILs in this study (1615-1810 $m \cdot s^{-1}$) were close to those observed by Sharma et al., which demonstrated similar chemical behavior in these compounds (1415-1566 $m \cdot s^{-1}$) [9].

Furthermore, extending the physical and chemical characterization of protic ionic liquids (PILs), the refractive indices of the compounds were evaluated to clarify the chemical interactions in the system (electrolyte/steel/PIL). Essentially, the electronic polarizability of molecules can be determined from their refractive index (n_D) (Table 1), which provides useful data about the compounds and helps to understand the interactive forces among molecules or their behavior in solution [3].

Indeed, Almeida et al. [10] reported interesting and clear data on the refractive index, which depends slightly on the volume of the anion in the solution. For example, in imidazolium-based ionic liquids, the integration of functional groups or an increase in the length of the alkyl group raises the electron density around the alkyl group [6]. Despite this, Deetlefs et al. [11] supported the relationship between molar volume and the increasing refractive index.

Thus, it is essential to add value to studies that thoroughly investigate atomic interactions in ionic liquids. However, the results obtained in some studies on protic and aprotic ionic liquid applications can differ from an electrochemical perspective, as a carbon chain from an acid can exert various interactions on the metallic surface, altering the basic or acidic nature of the electrolyte environment.

Therefore, as shown in Table 1, the average refractive index found in this work was approximately 1.4472 nD at 300 K, while the results reported by Sandar [3] were 1.51-1.54 nD at the same temperature. Likewise, Almeida [10] reported an average of 1.44-1.48 nD at 300 K, and S. Sardar [6] found an average of 1.49-1.55 nD. Thus, as the results above indicate, the close range of values confirms the importance of conducting the physical and chemical characterization of ionic liquids in general, whether they are aprotic or protic.

In fact, based on the moisture values presented in Table 1, ionic protic liquids are highly hygroscopic compared to other inhibitors, which corroborates the presence of a broad signal in the 4.3–4.8 ppm range in the hydrogen spectra, a characteristic of the water present in the sample (6.69%). This value is similar to the water content found by Viesca (2020) in their study (9.2% average), where the minimum water content was 0.6% and the maximum was 18.1%. Successive observations are essential to explain why the water content factor is not a significant issue for this application as a corrosion inhibitor. [8,10,12].

Nuclear Magnetic Resonance ^{1}H- NMR

Figures S1 and **S2** show the ^{1}H and ^{13}C NMR spectra of PILs A, B, and C, respectively. Their main structural modifications can be identified and used as evidence for the formation of the acid-base reaction due to the combination of diethanolamine and three different acids (formic, propanoic, and pentanoic acid), as reported in the literature [1,13,14].

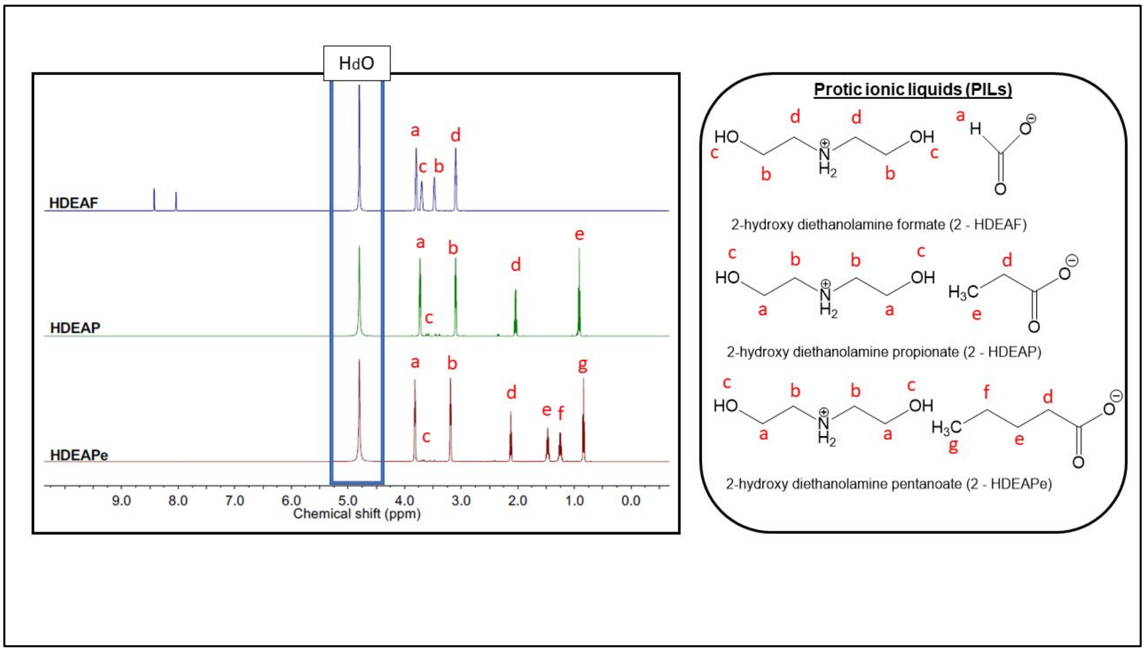

Fig. S1: ^{1}H NMR spectra of protic ionic liquids considered in the present study.

Nuclear Magnetic Resonance ^{13}C- NMR

Figure S2 confirms the ^{13}C NMR spectra of PILs A, B, and C, in which their main structural carbon compounds can be identified and used as evidence to support the formation of PILs.

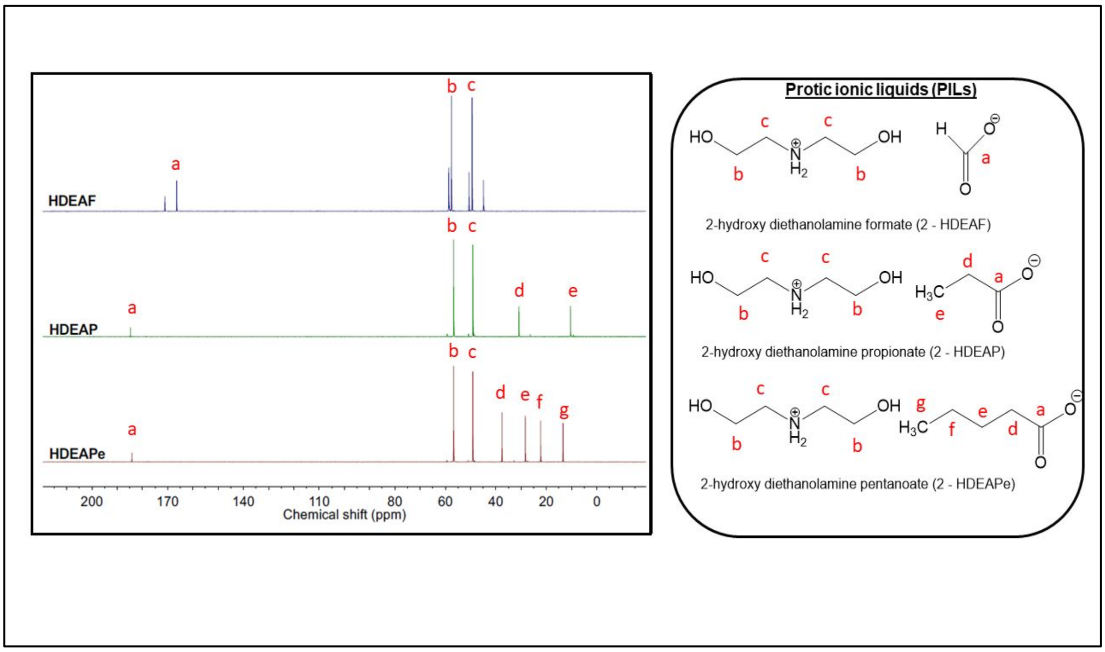

Fig. S2: ^{13}C NMR spectra of protic ionic liquids considered in the present study.

Fourier Transform Infrared Spectroscopy (FTIR)

Through FTIR analysis, it was possible to investigate the structural differences among the protic ionic liquids evaluated in this study. **Figure S3** shows the absorption spectra of PILs A, B, and C.

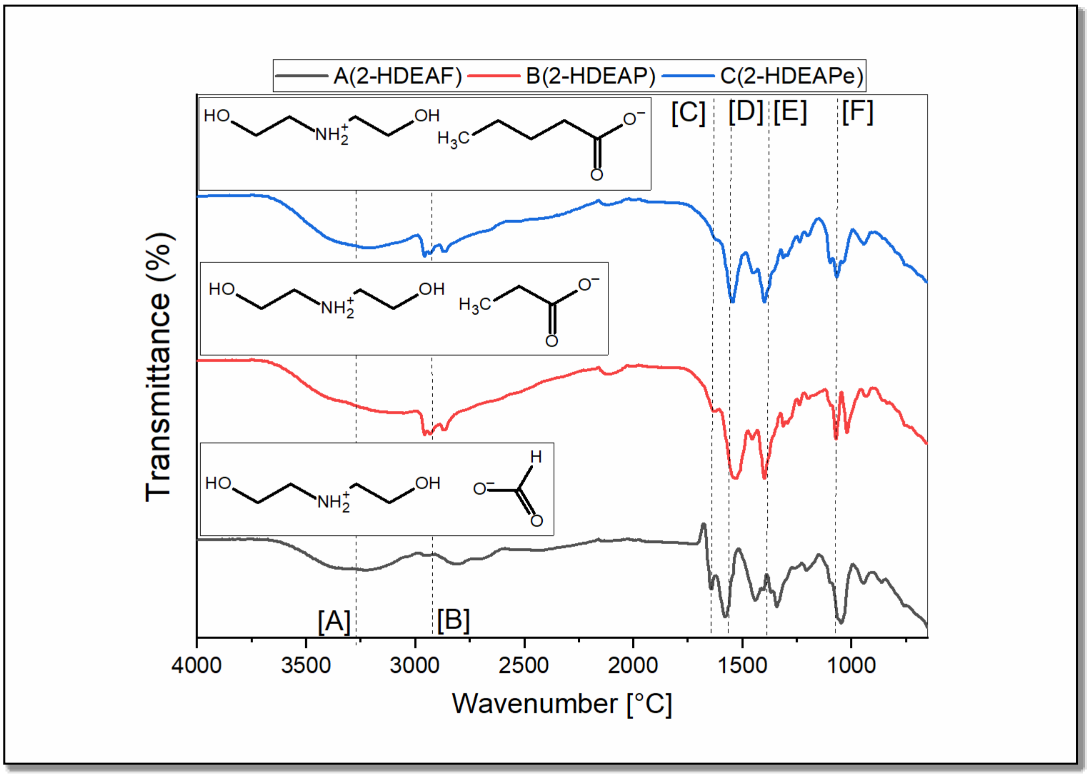

Fig. S3: FT-IR spectra of PIL A, PIL B, and PILC

A broad band, marked by sections A and B, in the range of 3600-2250 cm^{-1} presents the O-H and N-H stretching bands (3600-3200 cm^{-1}) and the C-H stretching band (3000-2800 cm^{-1}), which are characteristic of ammonium cation structures [1,13,14].

Furthermore, in the subsequent sections, C and D, two bands are noted at 1650 cm^{-1} and 1550 cm^{-1}, respectively. These bands can be attributed to the C=O asymmetric stretches of the anionic carboxylate and neutral carboxylate groups [1,13,14]. Finally, in the regions marked as E and F, bands at 1375 cm^{-1} are observed, which can be related to the symmetric angular deformation band of CH_2, and at 1090 cm^{-1}, which is attributed to the C-N stretching band.

Thermal Gravimetric Analysis (TGA)

Thermogravimetric analysis (TGA) is a technique regularly used to assess the thermal stability of chemical compounds, as thermal stability is typically described in terms of the onset temperature (T_{onset}), which is the crossing point between the two tangent lines on the TGA curve before and after material decomposition [3,14].

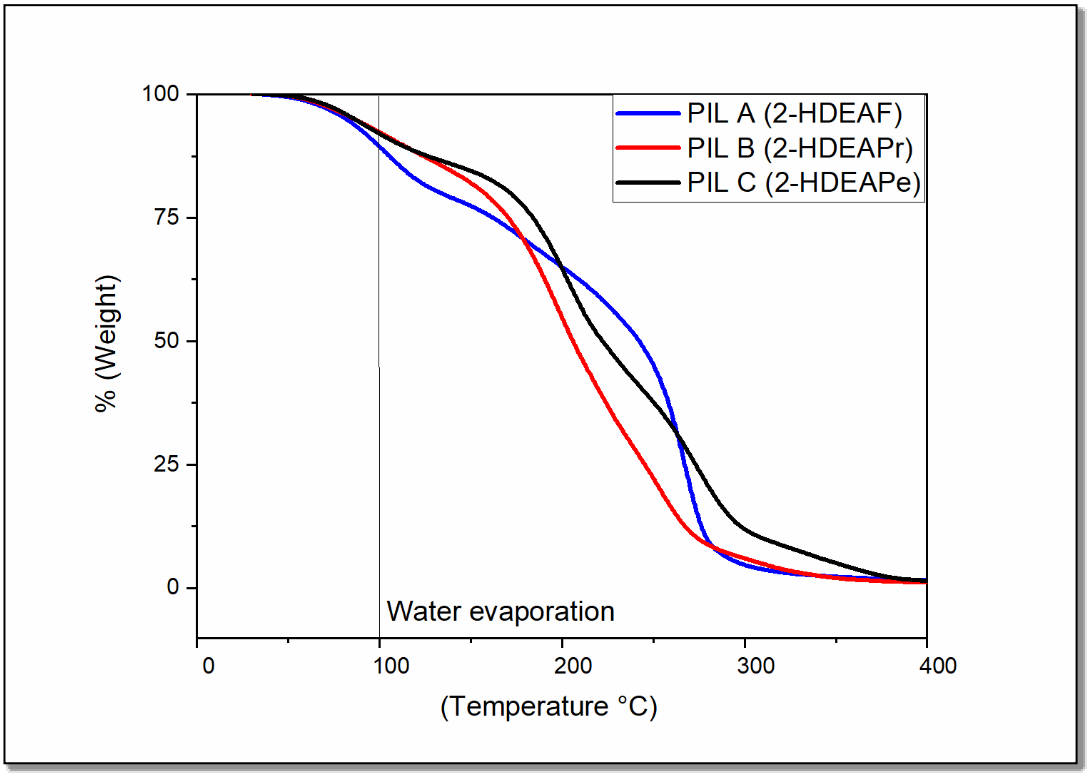

Fig. S4: Thermogravimetric analysis

Figure S4 shows a typical thermal decomposition curve for the three protic ionic liquids. It is observed that there is a plateau after the temperature range with the evaporation of water (100 °C), where the onset moment initiates at 160.25 to 171.45 °C, demonstrating that PILs have considerable thermal stability with respect to temperature increase. This represents a significant advantage compared to different types of green corrosion inhibitors, such as organic inhibitors, especially those that do not have considerable thermal stability, making them inapplicable when temperature factors are considered in a study. This study aims to ensure that in future work, the evaluation of corrosion inhibition activity can be related to different temperatures [13].

Table S2
Thermogravimetric temperature values obtained from PILs (PIL A, PIL B, and PIL C)

PIL	Onset	Tmáx
PIL A	241.2	265.5
PIL B	172.7	198.2
PIL C	179.1	204.9

Temperature in Celsius (°C)

Furthermore, as reported in the literature on ionic liquids, the addition of specific functional groups to the structure of a compound results in either a decrease or increase in thermal degradation [15–18]. Similar results were obtained in this study, considering that when carbon was added to the carboxylic acid main chain, a decrease of 10 °C was observed. This finding enhances our understanding because, in certain situations, smaller compounds in the ionic liquid can produce a more stable and stronger final product than larger ones in terms of the total number of atoms. This result can be observed from the values listed in **Table S2,** where the onset and T_{max} values are related.

References

(1) Bicak, N. A New Ionic Liquid: 2-Hydroxy Ethylammonium Formate. *J. Mol. Liq.* **2005**, *116* (1), 15–18. https://doi.org/10.1016/j.molliq.2004.03.006.

(2) Ohno, H. Functional Design of Ionic Liquids. *Bull. Chem. Soc. Jpn.* **2006**, *79* (11), 1665–1680. https://doi.org/10.1246/bcsj.79.1665.

(3) Sardar, S.; Wilfred, C. D.; Mumtaz, A.; Leveque, J. M.; Khan, A. S.; Krishnan, S. Physicochemical Properties, Brönsted Acidity and Ecotoxicity of Imidazolium-Based Organic Salts: Non-Toxic Variants of Protic Ionic Liquids. *J. Mol. Liq.* **2018**, *269*, 178–186. https://doi.org/10.1016/j.molliq.2018.08.017.

(4) David, M. *Applications of Ionic Liquids in Polymer Science and Technology*; 2015. https://doi.org/10.1007/978-3-662-44903-5.

(5) Hayyan, A.; Mjalli, F. S.; Alnashef, I. M.; Al-Wahaibi, T.; Al-Wahaibi, Y. M.; Hashim, M. A. Fruit Sugar-Based Deep Eutectic Solvents and Their Physical Properties. *Thermochim. Acta* **2012**, *541*, 70–75. https://doi.org/10.1016/j.tca.2012.04.030.

(6) Sardar, S.; Wilfred, C. D.; Mumtaz, A.; Leveque, J. M. Investigation of the Thermophysical Properties of AMPS-Based Aprotic Ionic Liquids for Potential Application in CO2 Sorption Processes. *J. Chem. Eng. Data* **2017**, *62* (12), 4160–4168. https://doi.org/10.1021/acs.jced.7b00552.

(7) Schmitzhaus, T. E.; Ortega Vega, M. R.; Schroeder, R.; Muller, I. L.; Mattedi, S.; Malfatti, C. de F. An Amino-based Protic Ionic Liquid as a Corrosion Inhibitor of Mild Steel in Aqueous Chloride Solutions. *Mater. Corros.* **2020**, *71* (7), 1175–1193. https://doi.org/10.1002/maco.201911347.

(8) Keshapolla, D.; Srinivasarao, K.; Gardas, R. L. Influence of Temperature and Alkyl Chain Length on Physicochemical Properties of Trihexyl- and Trioctylammonium Based Protic Ionic Liquids. *J. Chem. Thermodyn.* **2019**, *133*, 170–180. https://doi.org/10.1016/j.jct.2019.02.015.

(9) Sharma, G.; Gardas, R. L.; Coronas, A.; Venkatarathnam, G. Effect of Anion Chain Length on Physicochemical Properties of N,N-Dimethylethanolammonium Based Protic Ionic Liquids. *Fluid Phase Equilib.* **2016**, *415*, 1–7. https://doi.org/10.1016/j.fluid.2016.01.036.

(10) Almeida, H. F. D.; Lopes-Da-Silva, J. A.; Freire, M. G.; Coutinho, J. A. P. Surface Tension and Refractive Index of Pure and Water-Saturated Tetradecyltrihexylphosphonium-Based Ionic Liquids. *J. Chem. Thermodyn.* **2013**, *57*, 372–379. https://doi.org/10.1016/j.jct.2012.09.004.

(11) Deetlefs, M.; Seddon, K. R.; Shara, M. Predicting Physical Properties of Ionic Liquids. *Phys. Chem. Chem. Phys.* **2006**, *8* (5), 642–649. https://doi.org/10.1039/b513453f.

(12) Viesca, J. L.; Oulego, P.; González, R.; Guo, H.; Battez, A. H.; Iglesias, P. Miscibility, Corrosion and Environmental Properties of Six Hexanoate- and Sulfonate-Based Protic Ionic Liquids. *J. Mol. Liq.* **2021**, *322*, 114561. https://doi.org/10.1016/j.molliq.2020.114561.

(13) Guo, H.; Smith, T. W.; Iglesias, P. The Study of Hexanoate-Based Protic Ionic Liquids Used as Lubricants in Steel-Steel Contact. *J. Mol. Liq.* **2020**, *299*, 112208. https://doi.org/10.1016/j.molliq.2019.112208.

(14) Vega, M. R. O.; Parise, K.; Ramos, L. B.; Boff, U.; Mattedi, S.; Schaeffer, L.; Malfatti, C. F. Protic Ionic Liquids Used as Metal-Forming Green Lubricants for Aluminum: Effect of Anion Chain Length. *Mater. Res.* **2017**, *20* (3), 675–687. https://doi.org/10.1590/1980-5373-mr-2016-0626.

(15) Song, Y.; Xia, Y.; Liu, Z. Influence of Cation Structure on Physicochemical and Antiwear Properties of Hydroxyl-Functionalized Imidazolium Bis(Trifluoromethylsulfonyl)Imide Ionic Liquids. *Tribol. Trans.* **2012**, *55* (6), 738–746. https://doi.org/10.1080/10402004.2012.701000.

(16) Maton, C.; De Vos, N.; Stevens, C. V. Ionic Liquid Thermal Stabilities: Decomposition Mechanisms and Analysis Tools. *Chem. Soc. Rev.* **2013**, *42* (13), 5963–5977. https://doi.org/10.1039/c3cs60071h.

(17) Hao, Y.; Peng, J.; Hu, S.; Li, J.; Zhai, M. Thermal Decomposition of Allyl-Imidazolium-Based Ionic Liquid Studied by TGA-MS Analysis and DFT Calculations. *Thermochim. Acta* **2010**, *501* (1–2), 78–83. https://doi.org/10.1016/j.tca.2010.01.013.

(18) Cao, Y.; Mu, T. Comprehensive Investigation on the Thermal Stability of 66 Ionic Liquids by Thermogravimetric Analysis. *Ind. Eng. Chem. Res.* **2014**, *53* (20), 8651–8664. https://doi.org/10.1021/ie5009597.
